# Accelerated Amyloid Beta Pathogenesis by Bacterial Amyloid FapC

**DOI:** 10.1002/advs.202001299

**Published:** 2020-07-16

**Authors:** Ibrahim Javed, Zhenzhen Zhang, Jozef Adamcik, Nicholas Andrikopoulos, Yuhuan Li, Daniel E. Otzen, Sijie Lin, Raffaele Mezzenga, Thomas P. Davis, Feng Ding, Pu Chun Ke

**Affiliations:** ^1^ Australian Institute for Bioengineering and Nanotechnology University of Queensland Brisbane QLD 4072 Australia; ^2^ ARC Centre of Excellence in Convergent Bio‐Nano Science and Technology Monash Institute of Pharmaceutical Sciences Monash University 381 Royal Parade Parkville VIC 3052 Australia; ^3^ Department of Physics and Astronomy Clemson University Clemson SC 29634 USA; ^4^ Food & Soft Materials Department of Health Science & Technology ETH Zurich Schmelzbergstrasse 9, LFO, E23 Zurich 8092 Switzerland; ^5^ Interdisciplinary Nanoscience Center (iNANO) University of Aarhus Aarhus C Aarhus 8000 Denmark; ^6^ College of Environmental Science and Engineering Biomedical Multidisciplinary Innovation Research Institute Shanghai East Hospital Shanghai Institute of Pollution Control and Ecological Security Tongji University 1239 Siping Road Shanghai 200092 China; ^7^ Zhongshan Hospital Fudan University 111 Yixueyuan Rd, Xuhui District Shanghai 200032 China

**Keywords:** amyloid diseases, amyloidosis, brain health cross seeding, gut–brain axis, neurotoxicity

## Abstract

The gut–brain axis has attracted increasing attention in recent years, fueled by accumulating symptomatic, physiological, and pathological findings. In this study, the aggregation and toxicity of amyloid beta (A*β*), the pathogenic peptide associated with Alzheimer's disease (AD), seeded by FapC amyloid fragments (FapCS) of *Pseudomonas aeruginosa* that colonizes the gut microbiome through infections are examined. FapCS display favorable binding with A*β* and a catalytic capacity in seeding the peptide amyloidosis. Upon seeding, twisted A*β* fibrils assume a much‐shortened periodicity approximating that of FapC fibrils, accompanied by a 37% sharp rise in the fibrillar diameter, compared with the control. The robust seeding capacity for A*β* by FapCS and the biofilm fragments derived from *P. aeruginosa* entail abnormal behavior pathology and immunohistology, as well as impaired cognitive function of zebrafish. Together, the data offer the first concrete evidence of structural integration and inheritance in peptide cross‐seeding, a crucial knowledge gap in understanding the pathological correlations between different amyloid diseases. The catalytic role of infectious bacteria in promoting A*β* amyloidosis may be exploited as a potential therapeutic target, while the altered mesoscopic signatures of A*β* fibrils may serve as a prototype for molecular assembly and a biomarker for screening bacterial infections in AD.

## Graphical abstract

This study reveals a direct cross‐seeding linkage between bacterial amyloid protein FapC seeds and elevated A*β* fibrillization in vitro and in silico, and demonstrates the effects of such cross‐seeding on A*β*‐elicited neurotoxicity and Alzheimer's‐like pathology in a zebrafish model. This study points to the role of infectious bacteria in mediating brain health.

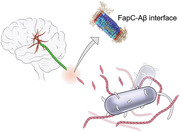

## 1 Introduction

The gut microbiome is essential for regulating the homeostasis in the body and plays subtle to pivotal roles in the pathogeneses of a wide range of human disorders, from dementia and inflammation to obesity, depression, and cancer.^[^ ^1^, ^2^, ^3^, ^4^ ^]^ The relevance of the gut–brain axis to human health has been proposed for more than a decade,^[^ ^5^ ^]^ and has recently come to the fore as a mainstream paradigm in the study of neurological disorders. Indeed, the discovery of the gut–brain axis has exposed a linkage between the gut microbiome, their metabolites, and neurological disorders such as Alzheimer's disease (AD) and Parkinson's disease (PD).^[^ ^6^, ^7^, ^8^ ^]^ Different anatomical and physiological connections, majorly from autonomic innervation of the gut, can communicate the influence of the gut microbiome toward the pathogenesis of neurological disorders.^[^ ^9^, ^10^, ^11^ ^]^ Ample evidence indicates that compromised gut‐microbiome can indirectly prompt the pathogenesis and progression of AD via maturation of autoimmune response against amyloid *β* (A*β*), BDNF expression, upregulation of NMDA receptors, and activation of proinflammatory cytokines like IL‐17A and NF‐kB.^[^ ^12^, ^13^, ^14^, ^15^ ^]^ On the other hand, alpha‐synuclein (*α*S), associated with PD, has been shown to shuttle from the gastrointestinal tract to the lower brain via the vagus nerve.^[^ ^16^, ^17^, ^18^ ^]^ A gut–brain neural circuit has been recently identified for nutrient and microglial sensory transduction, further implicating the gut microbiome as central to the signaling of the brain.^[^ ^19^, ^20^, ^21^ ^]^

AD is a major neurodegenerative disorder, characterized by the presence of extracellular A*β* plaques and intracellular hyperphosphorylated tau tangles.^[^ ^22^ ^]^ A*β* not only exists in the brain, but also in the circulation and in cerebrospinal fluid,^[^ ^23^ ^]^ raising the possibilities of its coaggregation and cross‐seeding with other amyloidogenic proteins.^[^ ^24^, ^25^ ^]^ Indeed, cytoplasmic A*β* and tau deposits have been found in the pancreatic *β* cells of AD and type 2 diabetes (T2D) subjects,^[^ ^26^ ^]^ and oligomeric and fibrillar A*β* and *α*S promoted each other's amyloidosis through in vitro cross‐seeding.^[^ ^27^ ^]^ Deposition of A*β* has induced enhanced cortical *α*S lesions in the brain autopsy of PD and Lewy body disease cases,^[^ ^28^ ^]^ while crossing transgenic (Tg) AD mice with *α*S Tg mice carrying an A53T mutation synergistically accelerated both AD and PD neuropathologies.^[^ ^29^ ^]^ Natural silk from *Bombyx mori*, prion protein Sup35 from *Saccharomyces cerevisiae*, and the bacterial curli protein CsgA from *Escherichia coli* all enhanced the amyloidosis of amyloid protein A (AA) in mice,^[^ ^30^ ^]^ suggesting that cross‐seeding proteins of distinctly different domains and pathogenic relevance is a plausible disease mechanism. Furthermore, curli stimulated aggregation of *α*S in biochemical assays as well as in vivo,^[^ ^31^ ^]^ and CsgC, which inhibited CsgA fibrillation both in vivo and in vitro, also inhibited aggregation of *α*S though not A*β*.^[^ ^32^ ^]^

**Scheme 1:**
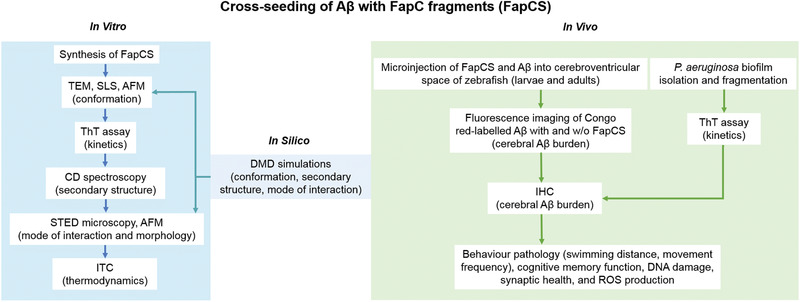
Cross‐seeding of A*β* and FapC fragments (FapCS), involving complementary in vitro and in silico techniques as well as in vivo assays.

While resemblances in sequence and aggregation state seemingly play a role, the molecular mechanisms of cross‐seeding amyloid proteins remain crucially lacking.^[^ ^33^, ^34^, ^35^ ^]^ As bacterial endotoxins and bacterial amyloid fragments possess realistic possibilities of accessing the brain via gastric autonomic innervation,^[^ ^9^, ^10^ ^]^ and crossing the compromised gut‐blood barrier and the blood‐brain barrier,^[^ ^36^, ^37^, ^38^ ^]^ especially for those having gut infections and/or aged population groups, here we examined the cross‐seeding capacity of FapC, a major protein constituent of the extracellular amyloid matrix of bacteria *Pseudomonas aeruginosa*, for A*β* (**Scheme** **1**). Our results collectively implicated that FapC seeds (FapCS) propagated their structural characteristics and acted as a catalyst for promoting A*β* amyloidogenesis in vitro, in silico and in a zebrafish AD model.

## 2 Results and Discussion

### 2.1 Scheme of Study

*P. aeruginosa*, though not a regular member of the physiological gut‐microbiota, is frequently involved in intestinal infections and inflammatory conditions like colitis, gastroenteritis, and diarrhea.^[^ ^39^, ^40^, ^41^ ^]^ *P. aeruginosa* secretes protein FapC, which self‐assembles into functional amyloid fibrils to constitute the biofilm scaffold.^[^ ^40^, ^42^ ^]^ The Fap operon is also shared by other opportunistic gastric pathogens like *Aeromonas caviae* and *Laribacter hongkongensis*.^[^ ^43^, ^44^ ^]^ To understand the gut–brain connection on the molecular level, here we first examined the cross‐seeding capacity of FapC fragments (obtained by sonication of mature fibrils to generate small seeds) on A*β* aggregation in vitro. In vivo cross‐seeding was studied by coinjecting FapC seeds (FapCS) and A*β* to the cerebroventricular space of larval and adult zebrafish. Pathological indicators such as behavior, cognitive memory function, cerebral A*β* burden, synaptic health, production of reactive oxygen species (ROS), as well as cell degeneration were examined. Finally, the biofilm from *P. aeruginosa* colonies containing FapC amyloid was isolated, fragmented and coinjected with A*β* fragments to the cerebrovascular space of adult zebrafish to study their compound pathologies (Scheme 1).

### 2.2 In Vitro Cross‐Seeding of FapCS and A*β*

**Figure 1:**
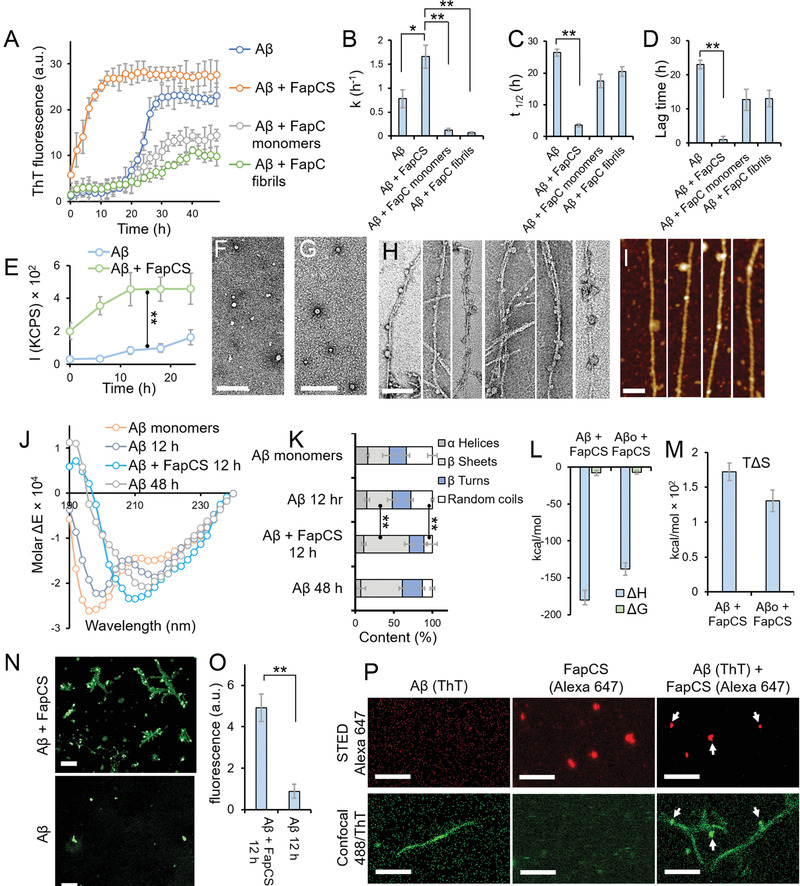
In vitro cross‐seeding between FapC fragments (FapCS) and A*β*. A) ThT assay of A*β* (50 × 10^−6^ m, incubated at 37 °C) in the presence or absence of FapCS, FapC monomers or preformed FapC fibrils (5 × 10^−6^ m) (*n* = 3). B‐D) ThT kinetic parameters of fibrillization rate constant *k* (B), half‐life *t*_1/2_ (C) and lag time (D) (*n* = 3). FapCS significantly increased (*, *p* < 0.05) *k* of A*β*, while the parameter was significantly suppressed (**, *p* < 0.005) with FapC monomer and fibrils. *t*_1/2_ and lag time of A*β* were significantly shortened (**, *p* < 0.005) by FapCS. E) Static light scattering (SLS) indicating a rapid growth in the size of A*β* fibrils, immediately after mixing with FapCS (*n* = 3). F) TEM images of A*β* (at 12 h showing prefibrillar species) and G) FapCS (scale bar: 100 nm). H) TEM and I) AFM images of A*β* incubated with FapCS at 12 h (scale bar: 100 nm). J) CD spectra and K) secondary structure of A*β* alone and with FapCS (*n* = 3). After 12 h of incubation, the negative peaks of A*β* at 215 and 198 nm were slightly increased and decreased in intensity, respectively, indicating a transition from random coil (decreased from 34 ± 6 to 28 ± 1.5%) to *β*‐sheet (slightly increased from 28 ± 9 to 33 ± 6%). At 48 h, a strong negative peak appeared at 201 nm representing formation *β*‐sheets rich (54 ± 4.5%) fibrils. However, similar *β*‐sheets rich structure (59 ± 6%) were observed in A*β* + FapCS sample at 12 h time point. L) Enthalpy (Δ*H*) and free energy (Δ*G*), M) entropic factor (*T*Δ*S*) for binding between FapCS and A*β* monomers or oligomers (A*β*o) (*n* = 3). N) Immunolabeling of A*β* with *β*‐amyloid specific antibodies (scale bar: 10 × 10^−6^ m). O) Quantification of green fluorescence intensity from (N) (*n* = 3). Significantly higher immune‐recognition (**, *p* < 0.005) was observed with A*β* + FapCS than A*β* alone, after 12 h incubation. P) STED microscopy of A*β* + FapCS (scale bar: 1 × 10^−6^ m). FapCS labeled with Alexa 647 appeared to be adsorbed onto or integrated into A*β* fibrils that were labeled with ThT.

FapCS was obtained by sonication of mature FapC fibrils (formed by 1 week of incubation of the protein in water, at 37 °C). The sonication settings (see Experimental Section) provided with finely dispersed FapCS seeds and maximum number of catalytic surfaces to interact with A*β*. Higher sonication power and time would have provided no notable differences and lower settings would increase the seed size and lower the availability of catalytic surfaces. Also, larger seeds could sequester A*β* on the longitudinal axis, decreasing the interaction with A*β* from the catalytic sites of the seeds. FapCS was then cross‐seeded with A*β* (50 ×10^−6^ m) at a 1:10 molar ratio, inducing an acceleration of A*β* fibrillization as indicated by a thioflavin T (ThT) kinetic assay (**Figure** **1**A). The rationale for using this molar ratio was to expose A*β* with a minimum amount of FapCS. As this ratio provided sufficient acceleration to bypass the lag phase of A*β* fibrillization, we opted for this ratio for the following experiments. In contrast, FapC monomers or fibrils lacked such acceleratory effect, suggesting the cross‐seeding capacity was specific to FapCS. This may be attributed to the high concentration of truncated ends in FapCS, which can stimulate the binding and fibrillization of A*β* monomers. In addition to that, the physical presence of FapC monomers and fibrils could reduce mutual A*β*–A*β* interactions to slow down the peptide fibrillization. Specifically, the fibrillization rate constant (*k*) of A*β* was markedly increased from 0.7 to 1.6 h^−1^ in the presence of FapCS (Figure 1B), while the lag time of A*β* was essentially abolished and the fibrillization half‐life was decreased by 86% (Figure 1C,D). Static light scattering (SLS) also reflected a significantly more rapid A*β* fibrillar growth when incubated with FapCS (Figure 1E). Transmission electron microscopy (TEM) imaging revealed that, when fibrillated alone, A*β* existed in the form of prefibrillary oligomers after 12 h of incubation (Figure 1F). A TEM image of FapCS is presented in Figure 1G. The molecular weight of FapC (PAO1) is 31.5 kDa,^[^ ^45^ ^]^ however during amyloid formation the protein molecules are tightly packed into the contour of their fibril.^[^ ^46^, ^47^, ^48^ ^]^ This can explain the formation of small‐sized spherical FapCS from mature and brittle FapC fibrils. In comparison, fully formed A*β* amyloid fibrils were observed in the A*β* + FapCS sample at 12 h (Figure 1H), where some FapCS appeared to adsorb onto the growing A*β* fibers. Atomic force microscopy (AFM) also displayed adsorption of FapCS on A*β* (Figure 1I), as well as further mesoscopic transformations of the A*β* fibrils upon cross seeding, as revealed by statistical analysis of the AFM images with FiberApp.^[^ ^49^ ^]^ Specifically, the pitch size of the A*β* fibrils was significantly shortened from 43 nm for native A*β* fibrils to 23 nm for A*β* fibrils seeded by FapCS, with the latter approximating that of FapC fibrils at 26 nm (Figure S1, Supporting Information). Furthermore, the A*β* fibrils seeded by FapCS possessed a diameter of 7.0 ± 2.1 nm, considerably thicker than that of native A*β* fibrils (5.1 ± 1.0 nm) or FapC fibrils (3.6 ± 1.0 nm) (Figure S1, Supporting Information, indicated accurately by fibril heights in AFM). This indicates that FapCS has major consequences on the mesoscopic properties of A*β* fibrils. The TEM images of A*β* incubated with FapC fibrils and monomers are presented in Figure S2 (Supporting Information). The FapC monomers appeared to aggregate on top of A*β* fibrils. A*β*, when incubated with preformed but nonsonicated FapC fibrils, did not coat FapC fibrils. However, the fibrillization of A*β* was significantly suppressed according to ThT fluorescence. In amyloidosis, physical properties like the pitch size and stiffness of amyloid fibrils evolve over time to yield increased fibrillar stiffness.^[^ ^50^, ^51^, ^52^ ^]^ Indeed, FapCS derived from younger FapC fibrils (fibrillated for 4 d at 37 °C and 25 °C) accelerated A*β* fibrillization (Figure S3, Supporting Information), but less than the seeds derived from mature FapC fibrils (fibrillated for 7 d at 37 °C) (Figure 1A). This age dependence can be explained by the brittle nature of mature fibrils,^[^ ^53^ ^]^ or simply by the greater population of mature fibrils to yield more seeds. In addition, A*β* seeds promoted an early onset of the peptide fibrillization as well (lag time: 1.9 h), but with a slower elongation than with FapCS (*k*: 0.03 h^−1^). This implies a lower seeding capacity of A*β* seeds than FapCS for the peptide aggregation (Figure S3, Supporting Information). In contrast to FapCS, the seeds from whey protein *β*‐lactoglobulin amyloids (bLgS) did not render any acceleration of A*β* fibrillization, as corroborated by ThT, SLS, and TEM assays (Figure S4, Supporting Information). The controls for the ThT and SLS assays are presented in Figure S5 (Supporting Information). A TEM image of control FapC and mature A*β* fibers is presented in Figure S6A (Supporting Information), while the ThT assay and the kinetic parameters of FapC alone are displayed in Figure S6B–E (Supporting Information). We further analyzed the secondary structural content of A*β*, in the presence or absence of FapCS, using circular dichroism (CD) spectroscopy (Figure 1J,K). The *β*‐sheet content of A*β* increased from 28% at 0 h to 33% at 12 h and 54% at 48 h, when incubated at 37 °C. However, coincubation of A*β* with FapCS resulted in an abrupt increase of the *β*‐sheet content to 59% at 12 h, indicating accelerated A*β* fibrillization (Figure 1K). In contrary to FapCS, bLgS did not induce *β*‐sheet formation in A*β*; instead the *α*‐helical and random‐coil structures of bLgS were slightly increased after incubation with A*β* indicating sequestration of A*β* monomers by bLgS (Figure S7, Supporting Information).^[^ ^54^ ^]^ Although both bLg and FapC are functional amyloids, the A*β* cross‐seeding was only observed with FapCS. This specificity of cross‐seeding with FapCS can be attributed to the intrinsic property of bacterial biofilms to dynamically communicate and cross‐seed, at inter and intra species level, to update their biofilms.^[^ ^55^, ^56^, ^57^ ^]^ The binding affinity between FapCS and A*β* monomers was further studied via isothermal titration calorimetry (ITC). The ITC data were corrected for A*β* against the diluent. The binding free energy (Δ*G* = −7.9 ± 3.5 kcal mol^−1^) indicated a weak but favorable interaction, in which a stabilizing (negative) contribution from enthalpy was partially cancelled out by a destabilizing (also negative) contribution from the entropic factor (*T*Δ*S* = −172 ± 12.4 kcal mol^−1^) (Figure 1L,M). The stabilizing enthalpic interaction between A*β* and FapCS could involve hydrogen bonding and electrostatic interaction. The stronger binding likely resulted in sequestration of A*β* and hence its aggregation mitigation, as verified by bLgS ( Figure S4A, Supporting Information). Insubstantial binding was observed for bLgS and A*β* by ITC (Figure S8, Supporting Information), which can be attributed to the non‐uniform nature of bLgS. Furthermore, we performed immunostaining of A*β* and A*β* + FapCS after 12 h of incubation (Figure 1N) using an antibody specific for A*β* fibrils. A*β* incubated with FapCS showed a much stronger antibody recognition than A*β* alone (Figure 1O), indicating faster A*β* fibrillization into immune‐recognizable amyloids in the presence of FapCS.^[^ ^58^ ^]^ Stimulated emission depletion (STED) microscopy, a super‐resolution imaging technique, revealed adsorption of FapCS (labeled with Alexa 647) onto A*β* amyloid fibrils (labeled with ThT) (Figure 1P). It can be deduced that, similar to TEM and AFM (Figure 1H,I), FapCS provided a catalytic surface through a mechanism similar to that of heterogeneous nucleation to initiate A*β* fibrillization.^[^ ^59^ ^]^ In addition to adsorption, intercalation of FapCS inside the A*β* fibrils was also observed (Figure 1P). This could occur when FapCS were smaller in size and were oriented in the right directions upon interacting with A*β* in fibrillization, as corroborated by the altered mesoscopic properties of A*β* fibrils upon cross seeding (Figure S1, Supporting Information). Finally, X‐ray diffraction on oriented fibrils revealed an intersheet and interstrand distance of 9.6 and 4.7 Å, respectively, comparable for native A*β*, FapCS‐seeded A*β* and FapC fibrils ( Figure S9, Supporting Information). The two distances were clearly separated into equatorial (9.6Å) and meridional (4.7Å) reflections as expected for a classic cross‐*β* structure.^[^ ^60^ ^]^

### 2.3 DMD Simulations Revealed the Molecular Interactions between FapCS and A*β*

**Figure 2:**
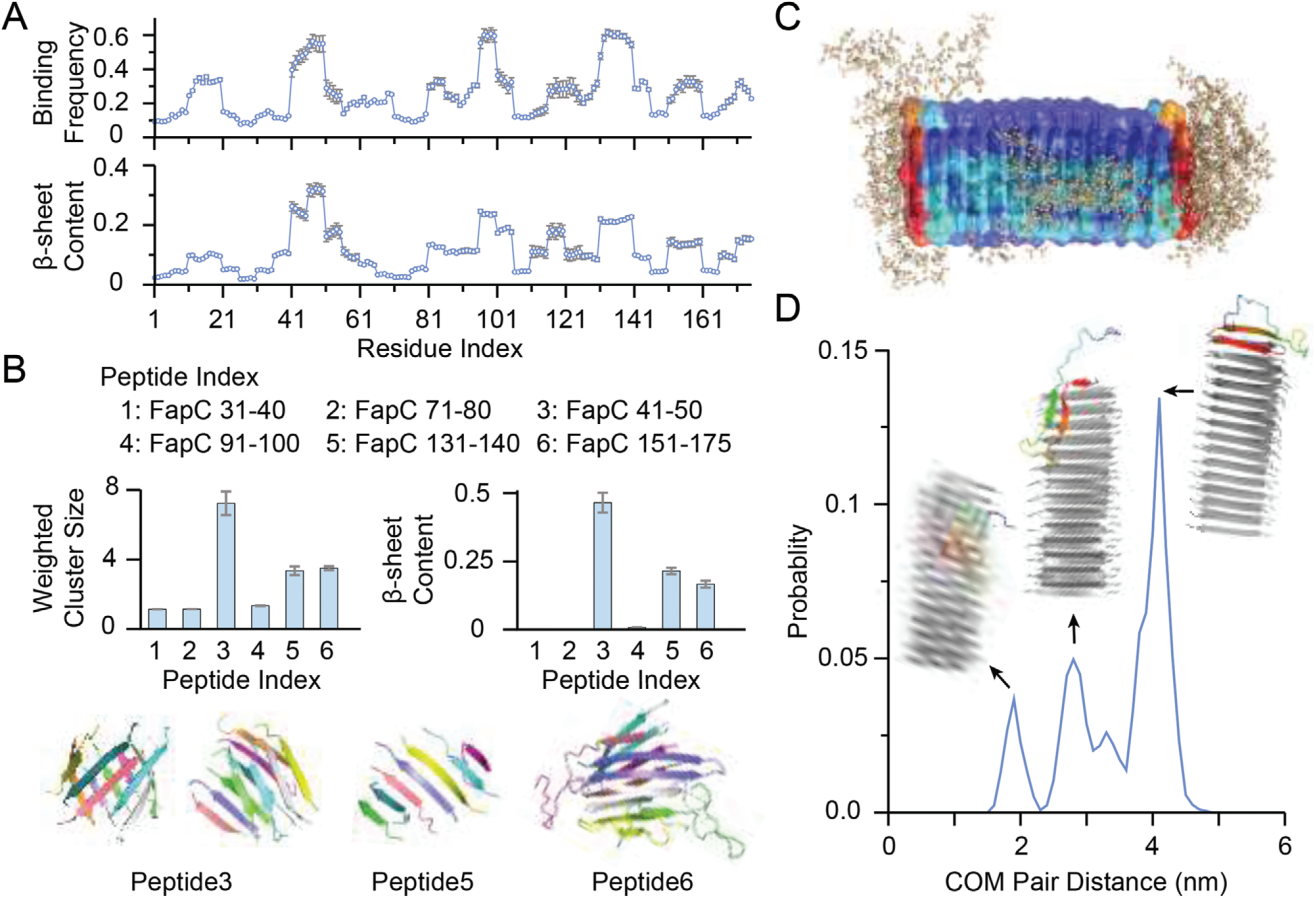
DMD simulations of molecular interactions between FapC and A*β*. A) The averaged binding frequency of each FapC with A*β*42 monomer (top) and the corresponding *β*‐sheet structure propensity (bottom). The results were obtained from binding simulations of A*β*42 with 10‐residue FapC with overlapping sequences. B) Mass‐weighted average cluster or aggregate sizes (top left) and *β*‐sheet contents (top right) obtained from aggregation simulations of six tested peptides, including three A*β*‐binding hotspot fragments and three controls for comparison. Typical snapshots of these cases formed *β*‐sheet rich aggregates (bottom). FapC was individually colored. C) Overlaying of final snapshots from ten independent cross‐seeding simulations, where preformed nanofibril by FapC41‐50 was shown as cartoon with molecular surface colored according to each residue's binding probability with A*β* from low (blue) to high (red). A*β*42 atoms were shown in wheat spheres. D) The intermolecular center‐of‐mass (COM) distance distribution between A*β*42 and the FapC nanofibril. Representative structures as cartoon were shown for each peak as inset with FapCS colored in gray and A*β*42 in rainbow.

We applied all‐atom discrete molecular dynamics (DMD) simulations—a rapid and accurate molecular dynamics algorithm^[^ ^61^ ^]^—to understand the molecular interactions between FapC and A*β*. This was done by mimicking the experimental identification of the binding hotspot regions between A*β* and human islet amyloid polypeptide in an earlier study.^[^ ^62^ ^]^ Briefly, the full‐length FapC sequence was divided into ten‐residue fragments with overlapping sequences, and binding simulations of each fragment with an A*β*42 monomer (abbreviated as A*β* hereafter) were performed (see Experimental Section). Three hotspot regions with residues 41–50, 91–100, and 131–140 were found to entail high binding frequencies with A*β* (**Figure** **2**A). These hotspot fragments tended to bind A*β* in the latter's central hydrophobic core of residues 16–22 and the C‐terminus 31–42, as indicated by the intermolecular contact frequency maps (Figure S10, Supporting Information). Driven by hydrophobic interactions, these peptides featured a relatively high *β*‐sheet content upon binding with A*β* (lower panel in Figure 2A).

To evaluate whether the A*β*‐binding hotspot fragments of FapC were amyloidogenic, the self‐assembly of multiple A*β* peptides was simulated. For comparison, three other nonhotspot fragments of residues 31–40, 71–80, and 151–175 were also tested. The C‐terminal 25‐residue fragment was selected from the repeat 3 region of FapC (R3), which is experimentally known to contribute to the fibrillization of FapC.^[^ ^63^ ^]^ For each 10‐residue fragment, ten peptides were used for the aggregation simulations while seven peptides were simulated for the 25‐residue R3 fragment. The hotspot fragment FapC41‐50 formed large aggregates with high *β*‐sheet contents, where both multilayer *β*‐sheets and *β*‐barrels were observed (Figure 2B). FapC131‐140 only formed small single‐layer *β*‐sheets, while FapC91‐100 stayed as monomers. For the nonhotspot fragments tested, only FapC150‐175 from the R3 region formed multilayer *β*‐sheets, in agreement with prior experimental studies.^[^ ^63^ ^]^

Since only FapC41‐50 among the A*β*‐binding hotspot regions was amyloidogenic, a preformed FapC41‐50 nanofibril was used to seed A*β* in the simulation. Starting from the dissociated state with random initial positions and orientations, A*β* bound the FapC nanofibril in all ten independent simulations. The nanofibril surfaces with strongest binding for A*β* were the two exposed ends with unsaturated backbone H‐bond donors and acceptors (Figure 2C). In most simulations, A*β* initially bound to the lateral surface and underwent random diffusion along the lateral surface towards the ends (Figure S11, Supporting Information), where both the central hydrophobic core around residues 16–22 and the C‐terminal residues 31–42 formed *β*‐sheets via H‐bonding with the exposed FapC backbone. Only in one out of the ten independent simulations A*β* stayed on the lateral surface of the FapC nanofibril. As a result, the probability distribution of center‐of‐mass (COM) distances between the A*β* monomers and the FapC41‐50 nanofibril featured three peaks (Figure 2D), where the highest peak corresponded to binding of the peptide at the two nanofibril ends. The other two minor peaks resulted from A*β* binding on the lateral nanofibril surface with the smallest intermolecular distances and the intermediate state with A*β* partially bound to the ends forming only one *β*‐strand with the nanofibril (Figure S11, Supporting Information). The simulation observations that the exposed FapC41‐50 nanofibril ends were more active than lateral surfaces in terms of A*β* binding and *β*‐sheet conversion were consistent with our experimental observation that FapCS, instead of FapC fibrils, accelerated A*β* aggregation (Figure 1A).

Our simulations demonstrated that FapC had several A*β*‐binding hotspots scattered along the protein sequence, among which only FapC41‐50 was amyloidogenic to allow incorporation of A*β* into the corresponding nanofibril, i.e., seeding the fibrillization of A*β*. These results were consistent with the experimental observation that a fraction of FapCS was incorporated into A*β* fibrils while other FapCS adsorbed to the sides of A*β* fibrils. Together, our computational and experimental results suggested that the exposed surfaces of FapCS upon sonication were likely heterogeneous and only a subset of FapCS with the “seeding‐competent” hotspots exposed could nucleate the formation of A*β* fibrils, while the rest bound to A*β* via other hotspot sequences.

### 2.4 FapCS Accelerated A*β* Pathology in Zebrafish Larvae

**Figure 3:**
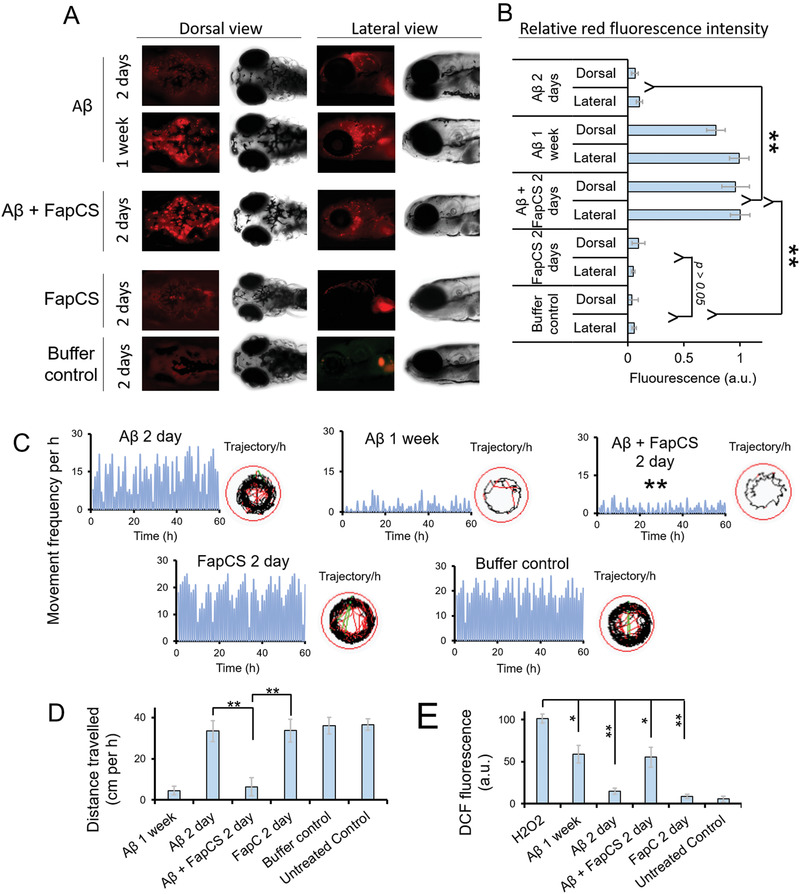
FapCS accelerated A*β* fibrillization and associated pathology in zebrafish larvae. A*β* (100 × 10^−15^ m), FapCS (10 × 10^−15^ m) or A*β* + FapCS at a 10:1 molar ratio (100:10 × 10^−15^ m) were injected to the cerebroventricular space of one week old zebrafish larvae. Congo red (100 × 10^−15^ m) was injected at 2 d or one week post A*β* injection to stain and monitor the A*β* fibrillization. A) The fluorescence images recorded via the brightfield and red fluorescence channel of a microscope for the whole‐mount dorsal and lateral side of larvae. B) Quantitative measurement of the relative red fluorescence intensity, indicative of Congo‐red‐stained A*β* plaques, from the cerebral region of larvae (*n* = 10). A*β* injected together with FapCS presented significantly stronger (**, *p* < 0.005) fluorescence and thus elevated A*β* fibrillization compared to A*β* alone or buffer, at 2 d postinjection. FapCS did not show any retention of Congo red fluorescence in the brain. C,D) A*β*‐induced behavioral pathology in terms of movement frequency (C) and total distance traveled by zebrafish larvae (D) (*n* = 10 per group and three groups per sample). The measurements were made for the observation period of 1 h at 2 d and one week postinjection. FapCS significantly aggravated (**, *P* < 0.005) A*β* toxicity and reduced the movement frequency and total travel distance of the larvae at 2 d postinjection. E) ROS generation in the brain homogenates of zebrafish larvae presented as relative DFC fluorescence. H_2_O_2_ was used as positive control. Similar to behavioral toxicity, A*β* + FapCS significantly enhanced ROS production in the larval brain (*n* = 10 per group and 3 groups per sample).

The zebrafish has recently been developed as a high‐throughput animal model to study A*β* toxicity and its associated AD pathologies.^[^ ^64^, ^65^ ^]^ Taking advantage of the optical transparency of zebrafish larvae, A*β* fibrillization after cerebroventricular injection can be monitored directly in the brain via Congo red.^[^ ^65^ ^]^ Here, one week old zebrafish larvae were injected with A*β* (100 × 10^−15^ m) or A*β* + FapCS at a 10:1 molar ratio. One week or 2 d postinjection, Congo red (100 × 10^−15^ m) was injected in the cerebroventricular space of the larvae and the presence of Congo‐red‐tagged A*β* amyloids in the larval brain tissues was imaged (**Figure** **3**A). Congo red is highly specific in staining of amyloid fibrils and is extensively used for fluorescence imaging of amyloid deposits in tissue samples.^[^ ^66^, ^67^, ^68^ ^]^ Higher levels of Congo red fluorescence (relative intensity: lateral/dorsal, 1/0.94), indicative of A*β* fibrillization, was observed 2 d postinjection with the sample of A*β* + FapCS (Figure 3B). However, it took one week for A*β* to fibrillate in the larval brain (relative intensity: lateral/dorsal, 0.98/0.68), while no fibrillization was observed 2 d postinjection (relative intensity: lateral/dorsal, 0.17/0.1). This indicates that FapCS was able to seed and effectively accelerate A*β* fibrillization in vivo. FapCS alone and the buffer control were unable to retain Congo red in the brain and insignificant fluorescence was observed at 2 d (Figure 3A) or one week time point (Figure S12A, Supporting Information). The neuronal toxicity of A*β* fibrillization was further evident in terms of behavioral pathology, i.e., movement frequency and distance traveled by zebrafish (Figure 3C,D). Specifically, A*β* coinjected with FapCS impaired the larval swimming behavior and the total distance traveled 2 d postinjection. The larvae injected with A*β* + FapCS swam 6.4 ± 4.4 cm h^−1^ as compared to 36 ± 4 cm h^−1^ for the buffer control. It took one week for A*β* alone to induce a similar extent of behavioral pathology. The slightly lower movement frequency for A*β* at 2 d can be attributed to the toxicity associated with A*β* fibrillization, which was insignificant at 2 d (first affecting movement frequency) but became more potent after one week. The larval movement frequency for the buffer control is presented in Figure S12B (Supporting Information). A*β* aggregation is associated with ROS, NO, and inflammatory cytokine generation via mitochondrial dependent or independent pathways to aggravate AD insults.^[^ ^69^, ^70^ ^]^ The ROS generation in the brain homogenates of larvae, treated with A*β*, A*β* + FapCS, or FapCS, was measured by a dichlorofluorescein (DCF) assay. The ROS induced by A*β* + FapCS 2 d postinjection (DCF fluorescence: 55.3 ± 11 a.u.) was comparable to that by A*β* alone at one week (59.1 ± 11 a.u.), and was significantly higher than that by A*β* alone at 2 days (8.9 ± 2.5 a.u.) (Figure 3E).

### 2.5 FapCS Accelerated A*β*‐Induced Cognitive Pathology in Adult Zebrafish

**Figure 4:**
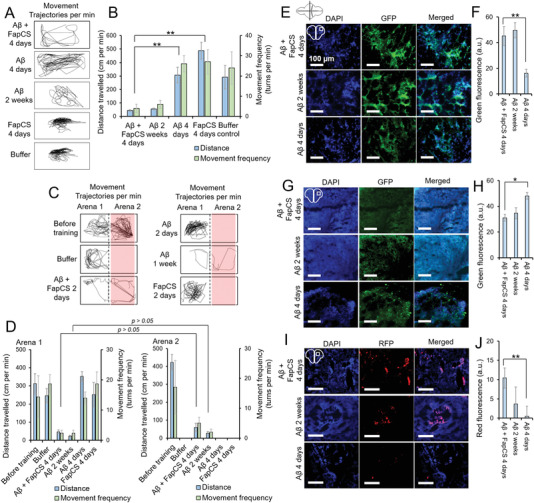
FapCS accelerated A*β*‐induced cognitive and neuronal pathology in adult zebrafish. A*β* (1 µL, 40 × 10^−6^ m) was injected to the cerebroventricular space of 10 months old adult zebrafish. A) Representative movement trajectories of adult fish after injection with FapCS, A*β*, or A*β* + FapCS. B) The quantitative measurements of movement frequency and total distance traveled by the fish (*n* = 3 per group and three groups per sample). A*β* + FapCS significantly suppressed (**, *p* < 0.005) the swimming behavior of adult fish, compared to A*β* alone at 4 d posttreatment. C) Cognitive memory function of the adult fish. The swimming tank was divided into arenas 1 and 2. Arena 2 was labeled with red paper from the bottom and fish was trained to avoid swimming into arena 2. The fish was shocked (9 V) whenever it swam into arena 2. D) Quantitative measurement of movement frequency and distance traveled in arena 1 versus arena 2. Before training, fish were able to freely swim in both arenas and no difference per arena was observed. After training, buffer injected and A*β* (4 d postinjection) fish were able to cognitively avoid swimming into arena 2. However, FapCS + A*β* and A*β* alone at 4 d and 2 weeks postinjection, respectively, were unable to avoid arena 2 and no significant difference in the swimming activities was observed in arena 1 versus arena 2 (*n* = 3 per group and 3 groups per sample). E) IHC for A*β* deposition in the brain of zebrafish. F) Quantitative measurement of green fluorescence for antibody staining (*n* = 3). G) Synaptophysin positive cells, H) quantitative measurement of anti‐synaptophysin antibodies labeling (*n* = 3), I) TUNEL assay and J) quantitative measurement for antibodies labeling in TUNEL assay (*n* = 3). A*β* alone at 2 weeks and A*β* + FapCS at 4 d presented a similar level of A*β* burden, neurodegeneration of synaptophysin positive cells and neuronal cell death in the fish brain, that were significantly different (**, *p* < 0.005) than A*β* alone at 4 d postinjection.

Zebrafish larvae offer a transparent in vivo model to characterize A*β* fibrillization and ROS generation for the study of A*β* neurotoxicity. Other advantages of this model include the feasibility of imaging A*β* deposition, allowing high‐throughput assays, and offering an in vivo vertebrate nervous system with all the protein machinery to study the neuropathology of AD. Quick development of A*β*‐toxicity phenotype (in 2–7 d) with small sample volumes is also an advantage of zebrafish as compared to rodent models.^[^ ^65^, ^71^ ^]^ However, zebrafish larvae are a developing organism, and hence are not optimal for probing the aging‐related clinical pathologies of AD.^[^ ^65^ ^]^ We therefore further studied the pathology of FapCS seeded A*β* in adult zebrafish that offer fully developed neuronal and behavioral systems that is clinically relevant to Alzheimer's pathologies.^[^ ^72^ ^]^ A*β* (1 µL, 40 × 10^−6^ m) alone or together with FapCS at a 10:1 molar ratio (A*β*:FapCS) was microinjected to the cerebroventricular space of adult zebrafish. The behavioral pathology, in terms of movement frequency and total distance traveled in a swimming tank, was monitored 4 d and 2 weeks postinjection (**Figure** **4**A,B). A*β* injected along with FapCS suppressed the zebrafish swimming behavior 4 d postinjection (movement frequency: 4 ± 2 per min, distance traveled: 41 ± 5.1 cm per min), which was insignificantly different to what was observed with A*β* alone 2 weeks postinjection (movement frequency: 6 ± 2 per min, distance traveled: 57 ± 6.9 cm per min). However, at 4 d postinjection A*β* did not produce significant difference in the swimming behavior compared to the buffer control (Figure 4B). Zebrafish express human orthologues for AD associated neuronal proteins, i.e., tau, *γ*‐secretase complex, presenilins and A*β* precursor orthologues, and adopt depression and anxiety associated behavior against environmental insults. This justifies the suitability of zebrafish as an animal model to study the clinically relevant AD pathology.^[^ ^73^, ^74^ ^]^ Therefore, we further assessed the impact of FapCS‐seeded A*β* on the cognitive memory function of adult zebrafish. The swimming tank was hypothetically divided into two halves (arenas 1 and 2), while one half (arena 2) was labeled with red paper and adult fish was trained to avoid arena 2 by electric shocks (dipping 9 V electrodes into the tank; see Experimental Section). After training, the buffer‐treated control fish avoided swimming into arena 2 (movement frequency: 0 per min, distance traveled: 0 cm per min) and retained their swimming activity within arena 1 (movement frequency: 18 ± 3 per min, distance traveled: 245 ± 38.9 cm per min) (Figure 4C). The fish injected with A*β* + FapCS were not able to avoid arena 2 at 4 d postinjection, and no difference was observed in their swimming activity in arena 1 versus arena 2 (Figure 4D). However, 4 d postinjection, A*β*‐injected fish were able to retain cognitive function and avoid swimming into arena 2. It took 2 weeks for the A*β*‐injected fish to produce a similar cognitively deficit behavior that was induced by FapCS + A*β* 4 d postinjection. FapCS alone did not induce any cognitive deficiencies.

We further performed immunohistochemistry (IHC) on the brain slices of adult zebrafish. A*β* deposition was significant in the brain of FapCS + A*β* treated fish 4 d postinjection, similar to the case of A*β* alone 2 weeks postinjection (Figure 4E,F). Two days postinjection A*β* alone did not render any deposition in the fish brain, corroborating the behavioral pathology data. In humans, A*β* deposition in the brain is associated with synaptic degeneration and neuronal cell death.^[^ ^75^, ^76^ ^]^ FapCS + A*β* and A*β* alone, at 4 d and 2 week postinjection respectively, were able to degenerate synaptophysin‐positive synapses and DNA damage per the TUNEL assay (Figure 4G–J). However, synaptophysin positive neuronal cells and negative TUNEL assay were observed in A*β*‐alone treated fish 4 days post injection. A*β* deposition, synaptophysin staining and TUNEL assay of FapCS alone and buffer‐treated control fish are presented in Figure S13 (Supporting Information). Healthy synapses and no DNA damage were observed with FapCS alone, indicating the non‐toxic nature of FapCS at 4 × 10^−6^ m of concentration.

### 2.6 *P. Aeruginosa* Biofilm Samples Elevated A*β* Pathology in Adult Zebrafish

**Figure 5:**
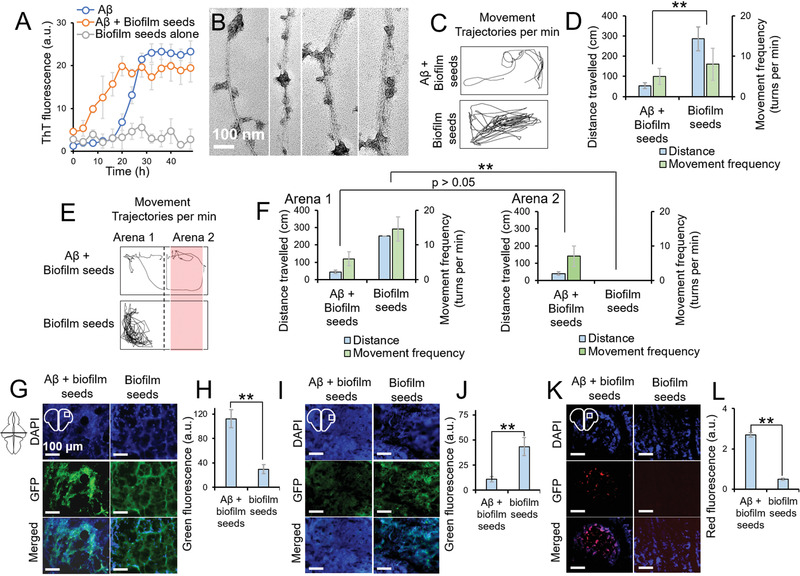
*P. aeruginosa* biofilm fragments elevated the A*β* pathology in adult zebrafish. ThT assay of A*β* (50 × 10^−6^ m, 37 °C) in the presence or absence of biofilm fragments (5 × 10^−6^ m, with respect to protein contents) (*n* = 3). Like FapCS, biofilm fragments significantly accelerated A*β* fibrillization. B) TEM images of A*β* fibrillized with biofilm fragments. The fragments appeared to be adsorbed onto A*β* fibrils. C) Movement trajectories and D) quantification of behavioral pathology, at 4 d postinjection, when A*β* was injected together with biofilm fragments (*n* = 3 per group and 3 groups per sample). Biofilm fragments enhanced A*β* toxicity and significantly (**, *p* < 0.005) suppressed the swimming behavior of adult zebrafish. E) Movement trajectory and F) quantification of the cognitive behavior of the fish in arena 1 versus arena 2 of the swimming tank (*n* = 3 per group and 3 groups per sample). Fish injected with biofilm fragments + A*β* presented deteriorated cognitive memory function at 4 d postinjection while biofilm fragments alone did not induce any behavioral toxicity. At 4 days postinjection, adult fish brains were subjected to IHC. H) A*β* deposition and I) quantification of A*β* deposition as green fluorescence of antibody labeling (*n* = 3), J) synaptophysin positive cells, K) quantification of antibody labeling of synaptophysin positive cells (*n* = 3), L) TUNEL assay and M) quantification of cell death in TUNEL assay (*n* = 3). Like FapCS, biofilm fragments enhanced A*β* deposition, accelerated the depletion of synaptophysin positive neurons and induced cell death in the brain of adult zebrafish.

To further demonstrate the cross‐seeding influence of bacterial biofilm samples on the pathogenesis of A*β*, we microinjected adult zebrafish with A*β* (1 µL, 40 × 10^−6^ m) and *P. aeruginosa* biofilm fragments (protein content) at a 10:1 molar ratio. The *P. aeruginosa* biofilms were isolated and fragmented via sonication. The biofilm fragments, like FapCS, accelerated A*β* fibrillization in vitro, as indicated by the ThT assay (**Figure** **5**A). The fibrillization rate constant decreased from 0.78 ± 0.19 to 0.45 ± 0.07 h^−1^. However, the lag time decreased more drastically from 23 h for A*β* to 2.4 h for A*β* + biofilm fragments (Figure S14, Supporting Information). Similar to FapCS, the biofilm fragments appeared to be adsorbed onto the growing A*β* fibrils (Figure 5B). TEM images of *P. aeruginosa* biofilms and their sonicated fragments are presented in Figure S15 (Supporting Information). The morphology of the biofilm seeds appeared to be different from FapCS, which can be attributed to the presence of other structural components in the bacterial biofilms.^[^ ^77^, ^78^ ^]^ Following the procedures with FapCS, we coinjected the biofilm fragments with A*β* to the cerebroventricular space of adult zebrafish. The biofilm fragments accelerated the A*β*‐induced behavioral and cognitive pathology 4 d postinjection, while the biofilm fragments themselves did not induce any toxicity (Figure 5C–F). Alongside the behavioral and cognitive dysfunctions, the biofilm fragments also aggravated A*β* deposition and caused damage to synaptophysin‐positive neurons and cell death, as indicated by IHC of the zebrafish brain slices (Figure 5G–L).

Although the zebrafish does not offer advanced cognitive learning and memory function, as with the rodent AD models, it offers direct imaging of A*β* deposition, quick disease modeling (2–7 d for larvae and 2 weeks for adult) with small sample volumes and neurobehavioral pathologies to study AD. These features can be attributed to the human orthologue genes for A*β*‐associated neuronal machinery and neurobehavioral physiology of zebrafish.^[^ ^65^, ^74^, ^79^, ^80^ ^]^

## 3 Conclusion

Amyloid fibrils are prone to fragmentation under agitation,^[^ ^81^ ^]^ and bacterial colonies along with their biofilms are under constant strain of peristalsis movement and enzymatic stress in the gut.^[^ ^82^, ^83^, ^84^ ^]^ In addition, bacterial amyloid proteins have the propensity to cross‐seed with other secreted proteins of the same colony,^[^ ^85^, ^86^ ^]^ and may translocate across the leaky gut–blood–brain barrier in aged or gut‐infected subjects^[^ ^87^ ^]^ or through autonomic innervation of the gut. Similarly, pathogenic amyloids can also cross‐seed, despite having different sequences, but following similar molecular mimicry of cross‐*β* stacking.^[^ ^24^, ^25^, ^27^, ^88^, ^89^ ^]^

The gut microbiome has been linked to neurological disorders and cancer via the metabolic and autoimmune pathways.^[^ ^90^, ^91^, ^92^ ^]^ In comparison, this study revealed a direct cross‐seeding linkage between pathogenic bacterial amyloid protein seeds and elevated A*β* fibrillization, neurotoxicity, and AD‐like pathologies in a zebrafish model. Structurally, FapC possessed several A*β*‐binding hotspots scattered along its sequence, with only one hotspot being amyloidogenic to allow the incorporation of FapC into A*β* nanofibrils via seeding. The exposed FapCS surfaces were heterogeneous, and therefore only a subset of FapCS exposing the “seeding‐competent” sequence of residues 41–50 could nucleate A*β* fibrillization. Accordingly, FapCS were incorporated into A*β* fibrils as well as adsorbed onto the A*β* fibril surface as observed experimentally (Figure 1). The seeding effects of FapC were further signified by the drastically altered morphology of A*β* fibrils in both pitch size and thickness. In addition to inducing structural integration and inheritance, as first revealed by this study, cross seeding with FapC further elevated the toxicity of A*β* in a zebrafish AD model, yielding severed behavioral and cognitive impairments.

In consideration of recent evidence of the gut–brain axis, together with the results from this study, it can be postulated that fragmented amyloids from infectious bacteria may cross‐seed pathogenic amyloidosis that underlies neurological disorders, thus playing a role in mediating brain health. The mesoscopic transformations of pathogenic amyloid fibrils resulting from cross seeding with bacterial amyloid proteins may stimulate the design of new biomolecular assemblies and facilitate clinical diagnosis of the causative origins of AD.

## 4 Experimental Section

##### Animal Husbandry and Ethics Statement

The wild‐type zebrafish (AB, *Danio rerio*) was bred in a circulatory system (Haisheng, Shanghai, China) at 28 ± 0.5 °C under a 14:10 h light and dark cycle. The experiments with the larvae were performed in Holtfreter's buffer. Tricaine (0.4%) in Holtfreter's buffer was used for anesthesia. For excision of the head from adult fish, the fish was placed in an ice‐chilled Holtfreter's buffer (0.4% tricaine) and the head was separated from the trunk using a sharp surgical knife. The excision was performed under a stereomicroscope and the heads were washed thrice in phosphate‐buffered saline (PBS, pH 7.4) and then stored in 2.5% paraformaldehyde. All in vivo experiments with zebrafish were performed according to Tongji University's ethical guidelines and the protocols were approved by the Animal Center of Tongji University (TJLAC‐019‐113). All other experiments, including in vitro, were performed according to the Occupational Health & Safety (OHS) guidelines of Monash University.

##### Synthesis of FapCS and Cross‐Seeding with A*β*

FapC seeds (FapCS) were produced by FapC fibrils of three different ages. The FapC monomers were produced as described^[^ ^45^ ^]^ and stored in 6 m GdmCl. Prior to fibrillization, the FapC monomers were purified via a PD10 desalting column. FapC monomers (1 mg mL^−1^ or 40 × 10^−6^ m) were incubated in deionized water at 37 °C for 1 week to produce mature fibrils and at 37 °C or 25 °C for 4 d to obtain young fibrils. Deionized water was used to make FapC fibrils as it is convenient to transfer the samples to other experimental conditions, i.e., buffered A*β* solution or PBS for in vivo microinjection. However, bacterial amyloids like FapC and curli can make amyloids over a range of conditions.^[^ ^93^ ^]^ FapCS were produced by sonicating an aqueous solution of FapC fibrils (10 × 10^−6^ m) at 20 power of a probe sonicator (Sonics Vibracell: 750 watts and 20 kHz) for 2 min with 1 s on/off cycle. For in vitro cross‐seeding experiments, 50 × 10^−6^ m of A*β* monomers were incubated with 5 × 10^−6^ m of FapCS (10:1 molar ratio, A*β*:FapCS) at 37 °C. bLg amyloids were synthesized by heating of bLg monomers (80 °C, pH 2) of 10 mg mL^−1^ solution for 24 h. Prior to the production of bLgS, the bLg amyloid solution was brought to pH 7 and then sonicated, similar to FapC, to generate bLgS.

*P. aeruginosa* biofilm fragments were generated by incubating fresh subcultures of bacteria (wild‐type reference strain PAO1) in cation‐adjusted Mueller‐Hinton broth (CAMHB) overnight in a 96‐well plate. After 48 h of incubation, the planktonic bacteria were pipetted out from the culture and replaced with fresh PBS (pH 7.4). The surface pellicle biofilm and ring biofilm attached to the well walls were scooped out and dispersed in PBS. The separated biofilm chunks were purified from the embodied bacteria by vortexing (10 min) with 20 µm glass beads and then repeated centrifugal washing (3 min, 2000 *g*) with PBS (pH 7.4). The biofilm containing supernatant was collected, concentrated, and adjusted to the concentration of 5 mg mL^−1^ in deionized water. The concentration was adjusted with respect to the protein contents of biofilm via the BCA assay. The biofilm fragments were subjected to sonication, same as FapC, to generate biofilm fragments.

##### ThT Kinetic Assay

A*β* (Anaspec Inc., purity ≥ 95%) was dissolved in 100 µL of hexafluoro‐2‐propanol (HFIP) and incubated at room temperature for 3 h. The acquired aliquots of this solution were freeze dried and stored at −20 °C for further experimentation. Before experiments, A*β* was dissolved in 0.1% NH_4_OH and then NH_4_OH was evaporated by opening the vial for 20 min. For the ThT kinetic assay, a 100 µL of aqueous solution containing A*β* (50 × 10^−6^ m), FapCS or bLgS (5 × 10^−6^ m) and ThT dye (100 × 10^−6^ m) was incubated (37 °C) in a 96‐well plate. The ThT fluorescence (excitation: 445 nm/ emission: 485 nm) was recorded at specified time intervals for 50 h. Kinetic parameters (rate constant *k*, half‐life *t*_1/2_ and lag‐time) were calculated as described.^[^ ^94^ ^]^ Briefly, kinetic curves were fitted to a sigmoidal curve $=y_{0}+\frac{y_{\max}-y_{0}}{1+e^{-(t-t_{1/2})k}}$, where *y*_0_ and *y*_max_ are initial and final ThT fluorescence values, allowing us to calculate lag time = *t*_1/2_ − 2/*k*.

##### Static Light Scattering (SLS)

Increases in the size of A*β* in the presence and absence of FapCS or bLgS were monitored by SLS. Solution of A*β* with FapCS or bLgS, at a similar ratio as for the ThT assay, was prepared and incubated. SLS measurements were performed at different time intervals using a Malvern Zetasizer Nano. The parameters were fixed at 7 for attenuator and 4.20 mm for cell position. Quartz cells with low volume capacity (100 µL) were used. The light scattering intensities (kilo counts per sec) and the refractive indices of the samples, buffer alone and toluene were measured.

##### Transmission and Atomic Force Microscopy

A*β* incubated in the presence or absence of FapCS, FapC monomers, FapC fibrils or bLgS were subjected to TEM imaging. After incubation for 12 h, a drop of sample was applied on a glow‐discharged carbon‐coated copper grid. After 1 min, the sample was blotted and then negatively stained with uranyl acetate (1%) for 30 s. The dried grid was then imaged with a Technei F20 TEM at 200 kV. For AFM, a drop of sample was placed on freshly cleaved mica surface and rinsed with water after 2 min and dried with pressurized air. The sample was scanned with an AFM (Nanoscope VIII Multimode Scanning Force Microscopes, Bruker), covered with an acoustic hood to minimize the vibrational noise. Images were acquired under the tapping mode with a silicon nitride cantilever (Bruker). AFM images were processed with Nanoscope Analysis 1.5 software to flatten the background and to calculate the statistical parameters.

##### Circular Dichroism Spectroscopy

The secondary structural contents of A*β* and A*β* fibrillized in the presence or absence of FapC or bLg species were determined by circular dichroism (CD) spectroscopy. Solutions of 200 µL, at similar molar ratios as for ThT assay, were prepared and incubated at 37 °C. CD spectra for different samples at 0, 12 and 48 h were measured with a CD spectrophotometer (Jasco) from 190 to 240 nm (1 nm step size). The percentage secondary structure was calculated by the CD data via Dichroweb analysis with parameters of Contin/reference set 4.^[^ ^95^ ^]^ For CD spectroscopy, A*β* was dissolved in 5 µL of 1% NH_4_OH buffer and then made up with deionized water for the rest of the experiment.

##### Isothermal Titration Calorimetry (ITC)

ITC (Malvern MicroCal iTC200) was used to explore the interaction between A*β* monomers and oligomers (A*β*o) with FapCS. Sample solutions were degassed at 25 °C while stirring (100 rpm). A*β* (200 × 10^−6^ m, 40 µL) was filled into the syringe while FapCS or bLgS (20 × 10^−6^ m, 200 µL) were filled in the cell (coin shaped). 0.1% NH_4_OH buffer solution was used for both solutions. A*β* was injected into the cell at an injection volume of 2 µL at the duration of 4 s and with a spacing of 180 s. First injection was 0.2 µL for 0.4 s duration and a total of 16 injections were programed. The heat exchange was recorded with a Microcal analysis (origin v7) software. The heat of dilution (A*β* against buffer) was subtracted from the interaction heat of samples. The acquired curves were fitted to a sequential binding model and thermodynamic parameters of binding energy (Δ*H*), free energy (Δ*G*), and entropic factor (*T*Δ*S*) were calculated. A*β*o for the ITC experiments was prepared by incubating A*β* aqueous solution (200 × 10^−6^ m) at 4 °C for 48 h.^[^ ^65^ ^]^

##### Stimulated Emission Depletion (STED) Microscopy

STED microscopy was employed to study the interaction of FapCS with A*β*. FapCS was labeled with Alexa 647 NHS ester via carbodiimide coupling reaction (1:1 molar ratio) in PBS (pH 8) for a duration of 5 h. The labeled fragments were purified via centrifugal filters (3000 kDa). Labeled FapCS was incubated with A*β* at the same molar ratio as for the ThT assay. After 12 h of incubation, the FapCS‐seeded A*β* fibrils were incubated with ThT dye (100 × 10^−6^ m) for 30 min to label the A*β* backbone and then purified via centrifugal filters (3000 kDa). A drop of 20 µL of the sample was placed on a poly‐l‐lysine coated coverslip, flipped on a glass slide and then imaged under STED microscope. Alexa and ThT fluorescence were imaged under the excitations of 647 and 488 nm and emissions of 665 and 525 nm, respectively.

For immune recognition of FapCS‐seeded A*β*, a drop of A*β* alone or A*β* + FapCS at 12 h incubation was placed and dried on a glass slide. The spot was washed with deionized water and then stained with primary and secondary anti‐A*β* antibodies as described in the IHC method. The labeled A*β* slides were imaged via the red channel of a fluorescence microscope.

##### Microinjection to Zebrafish Larvae and In Vivo A*β* Fibrillization

A*β* (HFIP‐treated) was dissolved in PBS (pH 7.4) to make a stock solution. A*β* (100 × 10^−15^ m) was injected into the cerebroventricular space of one week old zebrafish larvae. A*β* with FapCS was injected at a 10:1 molar ratio (A*β*:FapCS, 100 × 10^−15^:10 × 10^−15^ m). PBS alone and untreated larvae were used as negative controls. Zebrafish larvae were anesthetized by adding a few drops of tricaine (0.4%) in the petri dish. When larvae stopped to move in response to the tapping stimuli, the larvae were transferred to an injection template made from 1% agarose gel. Microinjection of A*β* was performed with a calibrated microneedle and pneumatic microinjection system (PV830 WPI), under a stereomicroscope. The injection pressure was set to 20 psi, the injection volume was 10 nL, and the injections were made with a micromanipulator. The tip of the needle was inserted into the ventricular space of larvae and penetration across the soft skin of dorsal tissue (central meeting point of both telencephalons) was not more than 0.3 mm. After microinjection, the larvae were transferred to the fresh Holtfreter's buffer in petri dish and incubated at 28 ± 0.5 °C for further observation.

To monitor A*β* fibrillization in the brain of zebrafish larvae, the A*β* or A*β* + FapCS injected larvae were further injected with Congo red 2 days or one week posttreatment. Congo red (100 × 10^−15^ m) was injected to the cerebroventricular space of the zebrafish larvae and placed back in Holtfreter's buffer (1 h) to allow staining of A*β* plaques in the brain tissues of larvae and elimination of excess dye. Whole mount imaging of zebrafish larvae was performed via the brightfield and red fluorescence channels of a fluorescence microscope. All images were captured under the same exposure and intensity settings of the microscope. Relative red fluorescence intensities were measured from the images by using Fiji ImageJ and represented as fraction of 1. A*β* at one week was considered as a positive control (100% or fraction 1) while buffer only was considered as a negative control (0% or fraction 0).

##### Behavioral Pathology and ROS Measurement in Zebrafish Larvae

Behavior of zebrafish larvae was observed by an automated zebrafish behavior recording system Zebrabox (Viewpoint). The behavior was characterized by total distance traveled (cm) and movement frequency, i.e., number of movements that were >90°, clock or anticlockwise. The trajectories of the zebrafish movements were also recorded by the instrument and all behavior measurements were done in a 96‐well plate with one larva per well. The observation period was 1 h and the number of larvae per group was 10 while three groups per sample were used. Untreated or buffer injected larvae were used as positive controls.

Reactive oxygen species (ROS) were measured by 2′,7′‐dichlorofluorescin diacetate (DCF). Zebrafish larvae were injected with samples as described for the behavior assay. At 2 d or one week posttreatment, the larvae were euthanized and heads of the larvae were separated from the trunks. For euthanizing, a few drops of concentrated (1%) tricaine were added to the petri dish of larvae. The truncated heads were collected, homogenized (70 Hz homogenizer for 5 min) and brought to an equal volume of 100 µL via PBS (pH 7.4). Then 100 µL of 2 × 10^−6^ m DCF was added to the homogenates and vortexed for 5 min in the dark. The DCF fluorescence was recorded with a microplate reader with excitation/emission of 495/529 nm. The number of larvae per group was 10 and three groups per sample were used to measure the ROS production.

##### Microinjection of Adult Zebrafish and Pathological Behavior

Adult zebrafish (10 months) were employed for the study. Prior to microinjection, adult zebrafish were anesthetized by placing them in a beaker filled with ice‐chilled tricaine (0.01% in Holtfreter's buffer) for 20 s. The samples of A*β* alone and A*β* with FapCS or bacterial biofilm fragments were injected to the cerebroventricular space of adult fish with a 1 µL Hamilton glass syringe. The concentration of the A*β* peptide was 40 × 10^−6^ m (in PBS, pH 7.4) while FapCS or biofilm fragments were included at a 10:1 ratio (A*β*:FapCS or biofilm fragments). The needle penetrated between left and right telencephalon for not more than 1 mm and injection volume was 1 µL. The syringe was washed with ethanol and PBS in between the injections. The injections were made under a stereomicroscope and fish were held in place with forceps. After microinjection, the fish was placed back in the water tank for recovery.

The behavioral pathology of the adult fish was observed after 4 d and 2 weeks of microinjection. The behavioral pathology of movement frequency and traveled distance was recorded with a zebrabox (Viewpoint) in a 1 L swimming tank. For cognitive function test, the tank was hypothetically divided into two parts of arena 1 and arena 2, and arena 2 was labeled by pasting a red paper at the bottom of the tank. The behavior of the untreated fish was recorded as positive control, in both arenas. Then fish from each sample were trained to avoid swimming in arena 2 by shocking with a 9 V battery electrodes, whenever the fish swim into arena 2. The fish were allowed to familiarize with the tank for 30 min and then trained for 20 min. After training, the swimming behavior of the fish was recorded in whole tank and then analyzed for arena 1 versus arena 2 with software EthnoVision X1.

The observations were made for 1 min with three fish per samples. The measurements were made three times in a day and then averaged together. The analysis parameters for the software were fixed as follows: animal: adult zebrafish, arena: open field square template (divided into two halves for the cognitive test); and the tracking features were set at central point, the sampling rate was 5 per s, the detection levels were sensitive enough to detect the fish in whole tank, and the threshold for movement frequency was 50° turn clock or counter clockwise and for travel distance was 0.5 cm. Movement trajectories were also recorded with the instrument's camera.

##### Immunohistochemistry (IHC)

The deposition of A*β*, depletion of synaptophysin positive cells and TUNEL assay were measured by IHC. The adult fish, at 4 d or 2 weeks postinjection with A*β* and A*β* with FapCS or biofilm fragments, were euthanized by placing in ice‐chilled tricaine (0.4% in Holtfreter's buffer) for 1 min. The head of the fish was separated at pectoral fin point with a sharp blade and fixed in paraformaldehyde as described in the “Animal husbandry and ethics statement” section. The fixed heads were dehydrated by dipping in gradual concentration of ethanol, i.e., 20%, 40%, 60%, 80%, and 100% with 10 min at each step. The heads were treated with 20% sucrose overnight, mounted with Tissue Trek OCT mounting medium at −20 °C. The heads were then sectioned into 20 × 10^−6^ m thick section with a cryostat. The sections were mounted on a gelatinized glass slide and immunostained with antibodies. The sections were dried, washed thrice in PBS (pH 7.4), and TritonX‐100 (0.05% in PBS) for 5 min each. The sections were dried and then a drop of primary antibody was placed on each section. Anti‐amyloid *β*42 (mouse monoclonal, Anaspec, AS‐55922) for A*β* deposition and antisynaptophysin antibody (Abcam, ab32594) for synaptophysin were used as primary antibodies. The slides were stored at 4 °C in a humidified chamber. After overnight incubation, the primary antibodies were washed with PBS and TritonX‐100 thrice and dried. A drop of secondary antibodies was placed on the sections and incubated for 4 h in a humidified chamber at 4 °C. Secondary antibodies of rabbit anti‐mouse IgG (H+L), HiLyte Fluor 488‐labeled (Anaspec AS‐28164‐05‐H488) for A*β* while goat antirabbit IgG H&L (Alexa Fluor 488) (Abcam, ab150077) were used for synaptophysin. For the TUNEL assay, Click‐iT Plus kit (Invitrogen C10619) was used. After incubation with secondary antibodies, the slides were washed with PBS and TritonX‐100 and dried. The concentration for primary and secondary antibodies was 1 µg mL^−1^ while DAPI at the concentration 1 µg mL^−1^ was included in secondary antibodies solution. The sections were imaged by a fluorescence microscope and fluorescence intensities were measured from the images with Fiji ImageJ analysis.

##### Discrete Molecular Dynamics Simulations

All‐atom DMD simulations with implicit solvent were applied to determine the hotspot regions in FapC that bound A*β*42, to evaluate the propensity of aggregation of various FapC fragments, and to test the cross‐seeding of A*β*42 by preformed FapC nanofibrils. All‐atom DMD simulations have been widely used^[^ ^96^, ^97^ ^]^ to study amyloid aggregation, including our recent study of FapC aggregation inhibition with nanosilver.^[^ ^98^ ^]^ Details of all‐atom DMD algorithms have been described in early studies.^[^ ^61^, ^99^ ^]^ To identify the A*β*‐binding hotspots in FapC, full‐length FapC was divided into 10‐residue peptides with five overlapping residues. For each 10‐residue fragment, twenty independent simulations were performed with an A*β*42 monomer starting with random intermolecular distances, orientations, and velocities. A cubic box with the periodic boundary condition and the dimension of 8 nm were used. Each independent simulation lasted 200 ns at room temperature 300 K. The final 150 ns simulation trajectories after reaching the steady states were utilized for data analysis. To investigate the amyloidogenic propensity of various FapC fragments, ten independent self‐assembling simulations were performed. Ten peptides, each of the following five sequences: FapC31‐40, FapC41‐50, FapC71‐80, FapC91‐100, FapC131‐140, and seven peptides, each of the 25‐residue sequence of FapC151‐175, were used in the aggregation simulations. A cubic box with a dimension of 12 nm was used, and each independent simulation lasted 350 ns at room temperature. The last 100 ns simulation trajectories after reaching the steady states were used for analysis. For the amyloidogenic and A*β*‐binding hotspot fragment FapC41‐50, an ideal double‐layer *β*‐sheet nanofibril with 34 peptides was built based on the zipperDB amyloid model of short peptides,^[^ ^100^ ^]^ which resembled the double‐layer *β*‐sheets from our self‐assembling simulations. The ideal preformed FapC fibril was then used for the cross‐seeding simulations with an A*β*42 monomer, where the nanofibril was kept static and A*β*42 was free to diffuse and undergo conformation changes. Ten independent 450 ns simulations were performed starting with the A*β*42 monomer randomly positioning away from the FapC41‐50 nanofibril in a 10.5 nm cubic box with the periodic boundary condition. The last 50 ns trajectories from all independent simulations were used for the analysis when the steady states were achieved. The interatomic distance cutoff of 0.65 nm was used to define an atomic contact. Two residues were considered in contact if they possess at least one intermolecular contact. Peptides were considered belonging to one aggregated cluster if they were all interconnected via single linkages. Protein secondary structures were defined according to the DSSP algorithm.^[^ ^101^ ^]^

##### Statistical Analysis

All experiments were repeated in triplicate, unless specified. Data are presented as mean ± standard deviation (SD). For zebrafish larvae experiments, 10 larvae per group and three groups per sample were used for measurement. For behavioral experiments with adults, three adult fish per samples were used and measurements were made three different times of a day. The significance was determined by one‐way ANOVA followed by Turkey's test and p values less than 0.05 (*) and 0.005 (**) were considered significant and highly significant, respectively. *p* value greater than 0.05 was considered insignificant. Data were analyzed via SPSS Statistics.

## Conflict of Interest

The authors declare no conflict of interest.

## Author Contributions

I.J. and P.C.K. conceived the project. I.J., P.C.K., and Z.Z. wrote the manuscript. I.J. performed seed preparation, cross‐seeding, ThT, TEM, CD, ITC, in vivo microinjection, adult and larval zebrafish behavioral, cognitive, ROS and immunostaining assays and data analyses. N.A. and Y.L. conducted the ThT kinetic assay on A*β* self‐seeding. J.A. and R.M. performed AFM and data analysis. Z.Z. and F.D. performed DMD simulation studies. D.O. provided reagents and edited the manuscript. All authors agreed on the presentation of the manuscript.

## Supporting information

Supporting Information
