# Supplementary material for: Accelerated Amyloid Beta Pathogenesis by Bacterial Amyloid FapC
Source: Adv Sci (Weinh). 2020 Jul 16;7(18):2001299. doi: 10.1002/advs.202001299 (PMC7509637; doi:10.1002/advs.202001299)
Supplement: Supplementary file 1 — Supporting Information [file ADVS-7-2001299-s001.pdf]

Supplementary Information

Accelerated Amyloid Beta Pathogenesis by Bacterial Amyloid FapC

Ibrahim Javed,[1,2] Zhenzhen Zhang,[3] Jozef Adamcik,[4] Nicholas Andrikopoulos,[2] Yuhuan Li,[2] Daniel E. Otzen,[5] Sijie Lin,[6] Raffaele Mezzenga,[4] Thomas P. Davis,[1,2] Feng Ding[3*] and Pu Chun Ke[7,2*]*

[1]Australian Institute for Bioengineering and Nanotechnology, University of Queensland, Brisbane Qld 4072, Australia

[2]ARC Centre of Excellence in Convergent Bio-Nano Science and Technology, Monash Institute of Pharmaceutical Sciences, Monash University, 381 Royal Parade, Parkville, VIC 3052, Australia

[3]Department of Physics and Astronomy, Clemson University, Clemson, SC 29634, USA

[4]Food & Soft Materials, Department of Health Science & Technology, ETH Zurich, Schmelzbergstrasse 9, LFO, E23, 8092, Zurich, Switzerland

[5]Interdisciplinary Nanoscience Center (iNANO), University of Aarhus, 8000 Aarhus C, Denmark

[6]College of Environmental Science and Engineering, Biomedical Multidisciplinary Innovation Research Institute, Shanghai East Hospital, Shanghai Institute of Pollution Control and Ecological Security, Tongji University, 1239 Siping Road, Shanghai 200092, China

[7]Zhongshan Hospital, Fudan University, 111 Yixueyuan Rd, Xuhui District, Shanghai, 200032, China

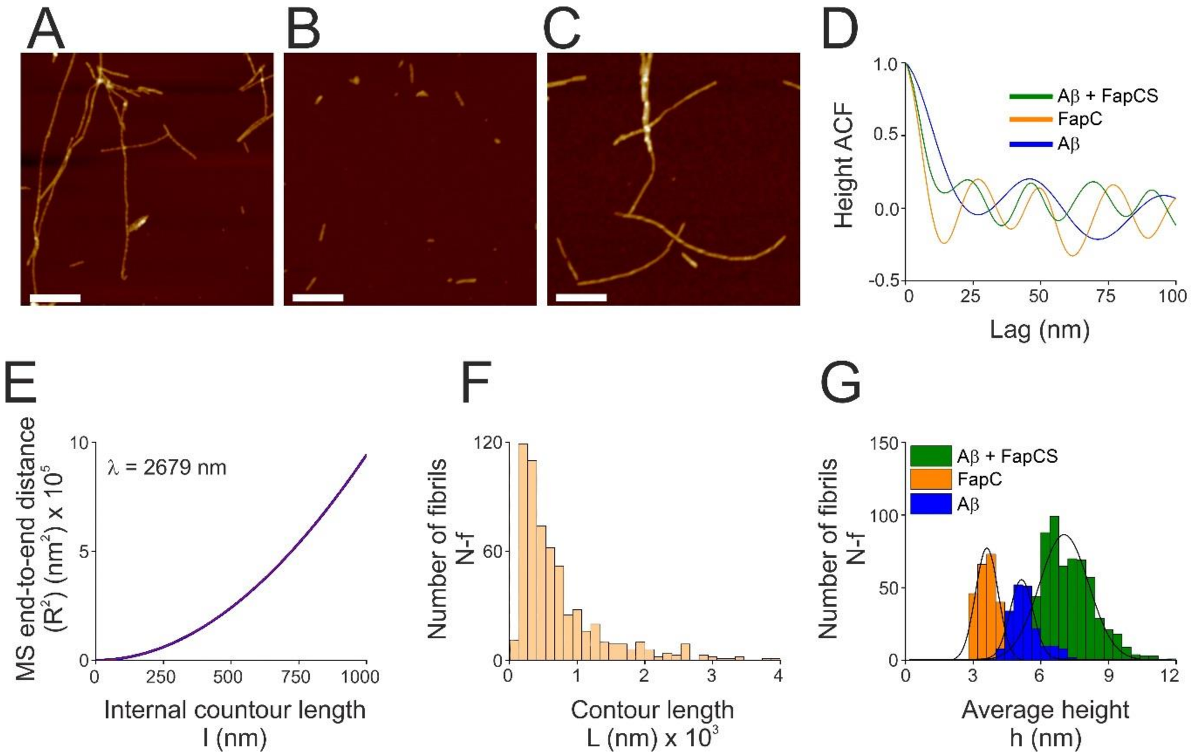

Supplementary Figure 1. AFM and morphological statistics for FapCS-seeded Aβ, Aβ and FapC fibrils. AFM images of (A) mature FapC fibrils (37 ºC, 1 week), (B) FapCS and (C) Aβ fibrils. Scale bar for all AFM images is 200 nm. (D) Estimation of periodicity of FapCS-seeded Aβ, Aβ and FapC fibrils calculated from the autocorrelation function (ACF) of AFM images. (E) Mean squared end-to-end distance versus internal contour length providing the persistence length of FapCS-seeded Aβ fibrils. (F) Contour length distribution of FapCS-seeded Aβ fibrils. (G) Average height distributions of FapCS-seeded Aβ, FapC and Aβ fibrils.

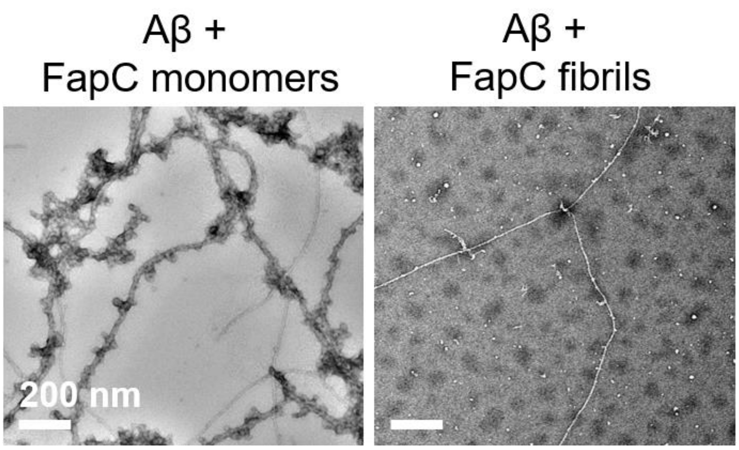

Supplementary Figure 2. TEM images for Aβ fibrillized in the presence of FapC monomers and preformed FapC fibrils. Thick Aβ fibrils coated with FapC monomers were observed under TEM. Incubation time was 50 h at 37 °C. Aβ, when incubated with

preformed FapC fibrils, did not coat FapC fibrils. However, the fibrillization of Aβ was significantly suppressed.

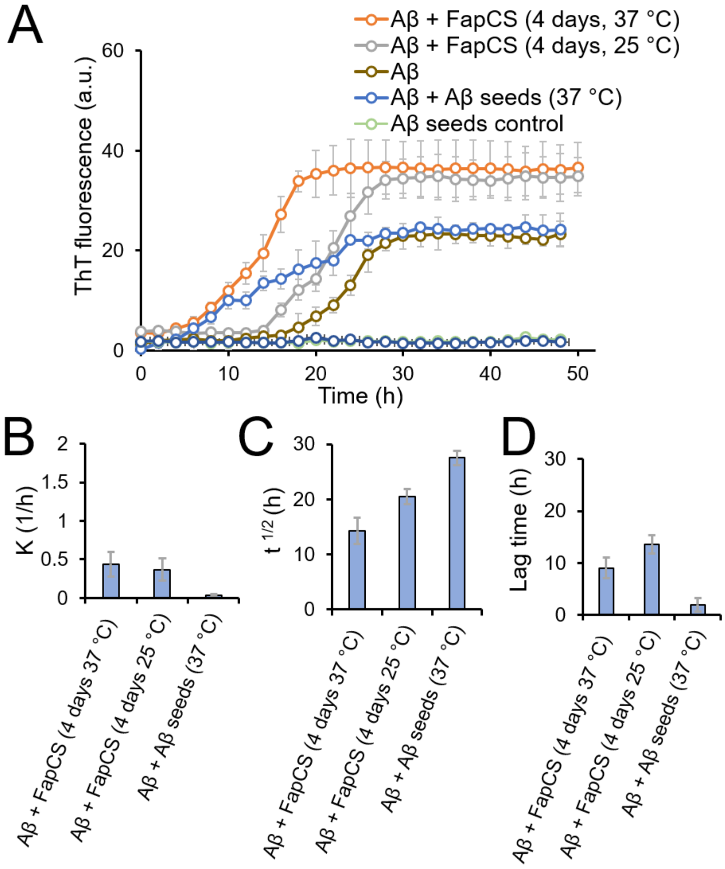

Supplementary Figure 3. ThT kinetic assay and parameters of Aβ with FapCS from younger FapC fibrils, with Aβ seeds. FapCS (5 μM) from younger FapC fibrils (fibrillized at 37 and 25 °C for 4 days) also accelerated Aβ (50 μM) fibrillization, however, not to an extent observed for FapCS from mature FapC fibrils (fibrillized at 37 °C for 1 week) (n=3). Self-seeded Aβ (Aβ + Aβ seeds, 10:1 molar ratio) shortened the lag phase to 1.9 h from 23 h for Aβ alone while the fibrillization rate constant *k* is decreased to 0.03 h^{-1}.

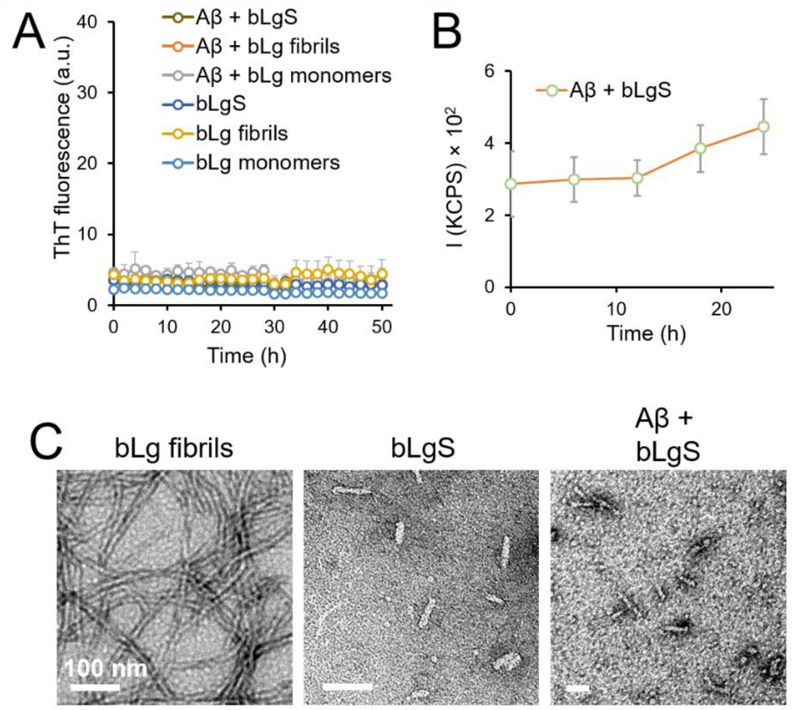

Supplementary Figure 4. Seeds from functional amyloids of β-lactoglobulin (bLgS) and their impact on Aβ fibrillization. bLgS were produced via sonication and were cross-seeded with Aβ. (A) ThT assay indicated that bLgS, bLg monomers and preformed amyloids (5 μM) inhibited Aβ (50 μM) fibrillization (n=3). (B) SLS of Aβ fibrillized in the presence of bLgS (n=3). (C) TEM micrographs of bLg fibrils, bLgS and Aβ + bLgS.

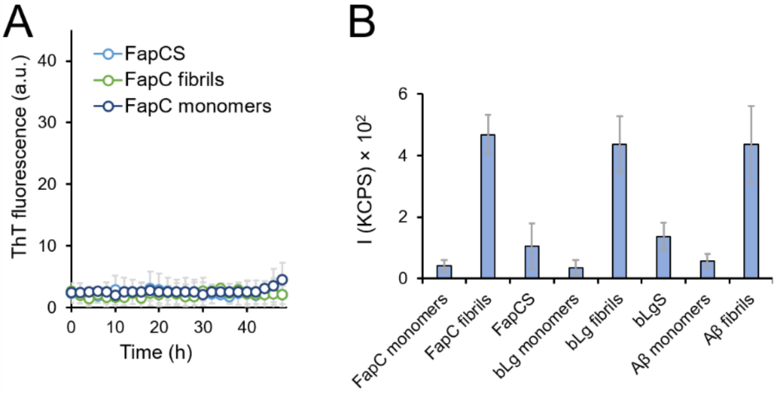

Supplementary Figure 5. (A) ThT assay of FapCS, FapC fibrils and FapC monomers (5 μM), as controls, for 50 h incubation at 37 °C (n=3). (B) SLS of controls of FapC monomers and fibrils, FapCS, bLgS, bLg monomers and fibrils, and Aβ monomers and fibrils (n=3).

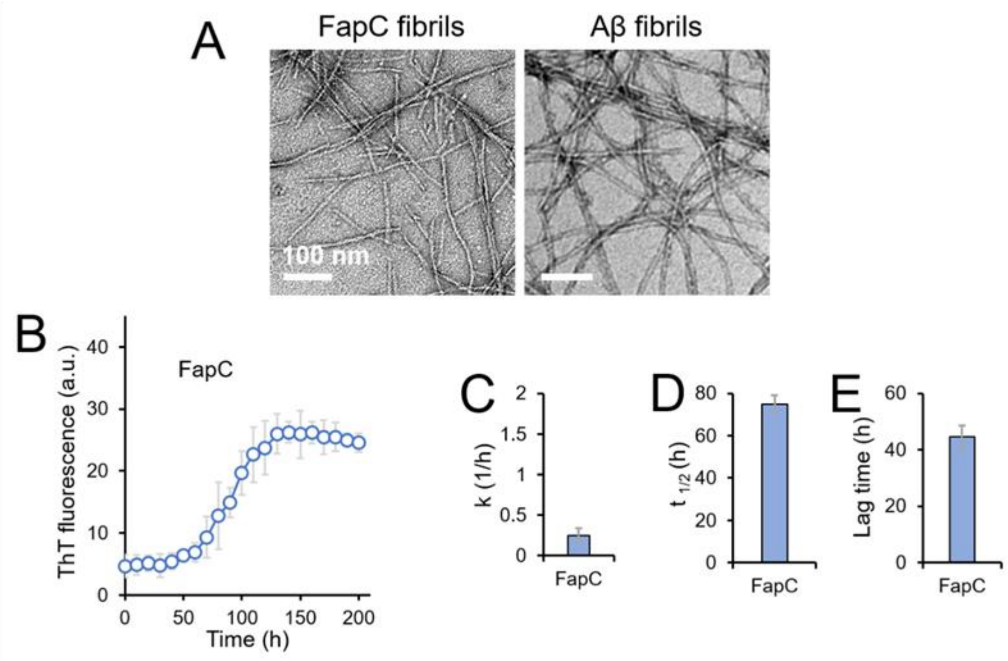

Supplementary Figure 6. (A) TEM of FapC and Aβ fibrils. (B) ThT assay of FapC (50 μM) at 37 °C (n=3). It took 150 h for FapC to reach the saturation phase. (C) Fibrillization rate constant k, (D) half-life $t_{1/2}$ and (E) lag time of FapC fibrillization (n=3).

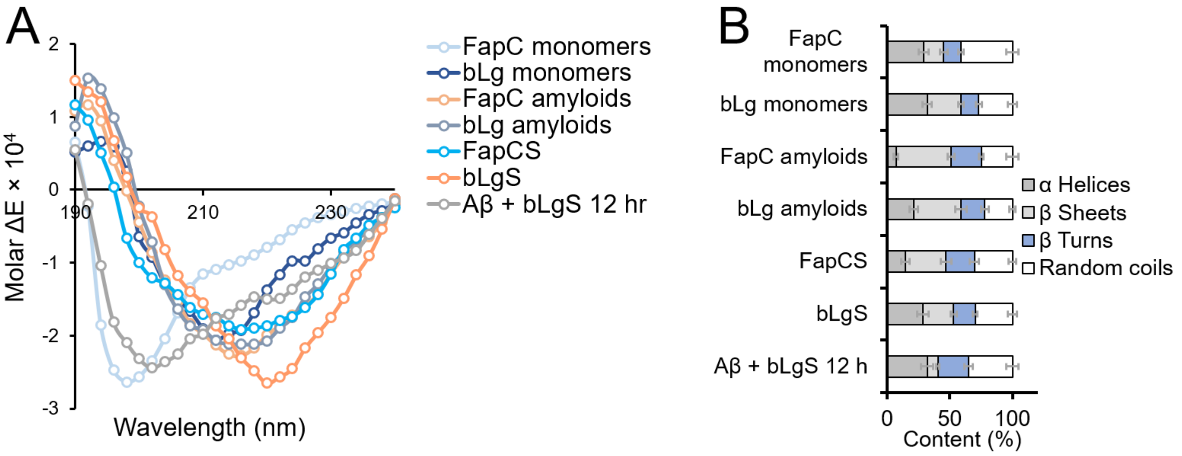

Supplementary Figure 7. Secondary structure of FapC and bLg controls and Aβ in the presence of bLgS. (A) CD spectra and (B) secondary structure (%) of monomers and fibrils of bLg and FapC, bLgS, FapCS and Aβ in the presence of bLgS at 12 h incubation at 37 °C (n=3). After incubation with Aβ, the α-helical content of bLgS was slightly increased.

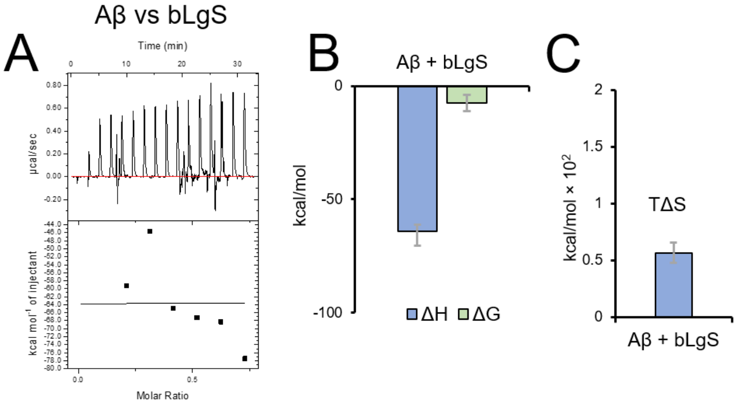

Supplementary Figure 8. (A) Isothermal titration calorimetry (ITC) of Aβ with bLgS. (B) Binding enthalpy (*ΔH*) and free energy (*ΔG*) and (C) entropic factor (*TΔS*) of Aβ with bLgS (n=3).

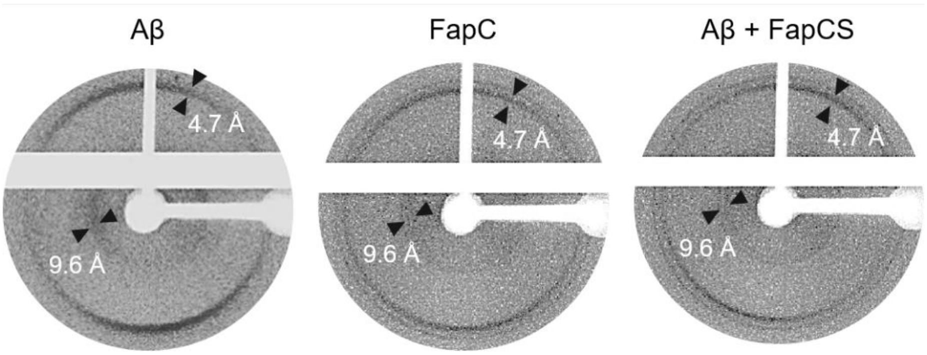

Supplementary Figure 9. X-ray fiber diffraction of Aβ, FapC and Aβ + FapCS. All three samples showed similar inter-sheet and inter-strand distance of 9.6 and 4.7 Å, respectively.

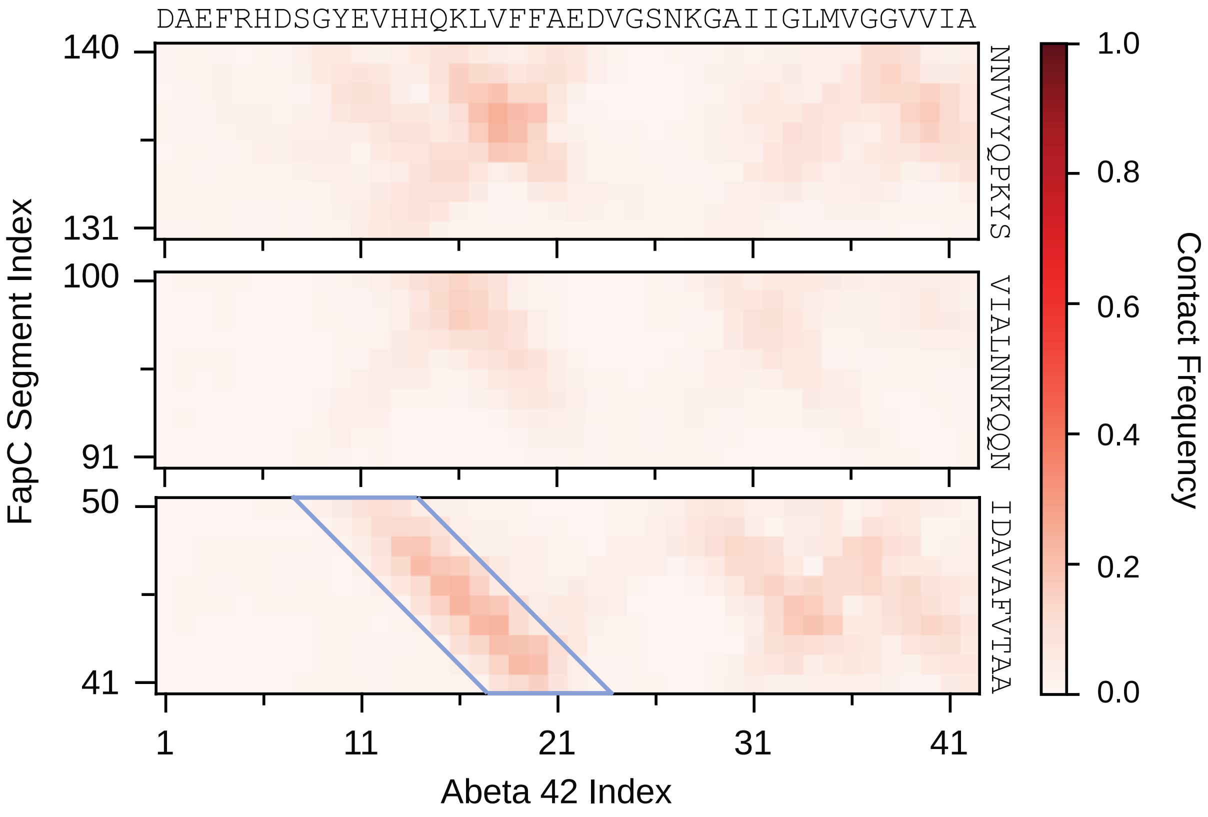

Supplementary Figure 10. The inter-molecular contact frequency maps for the binding of three hotspot fragments (FapC41-50, FapC91-100, FapC131-140) with an Aβ42 monomer. Anti-parallel β-sheet formed between FapC41-50 and the amyloidogenic region of Aβ16-22 is highlighted with a blue box.

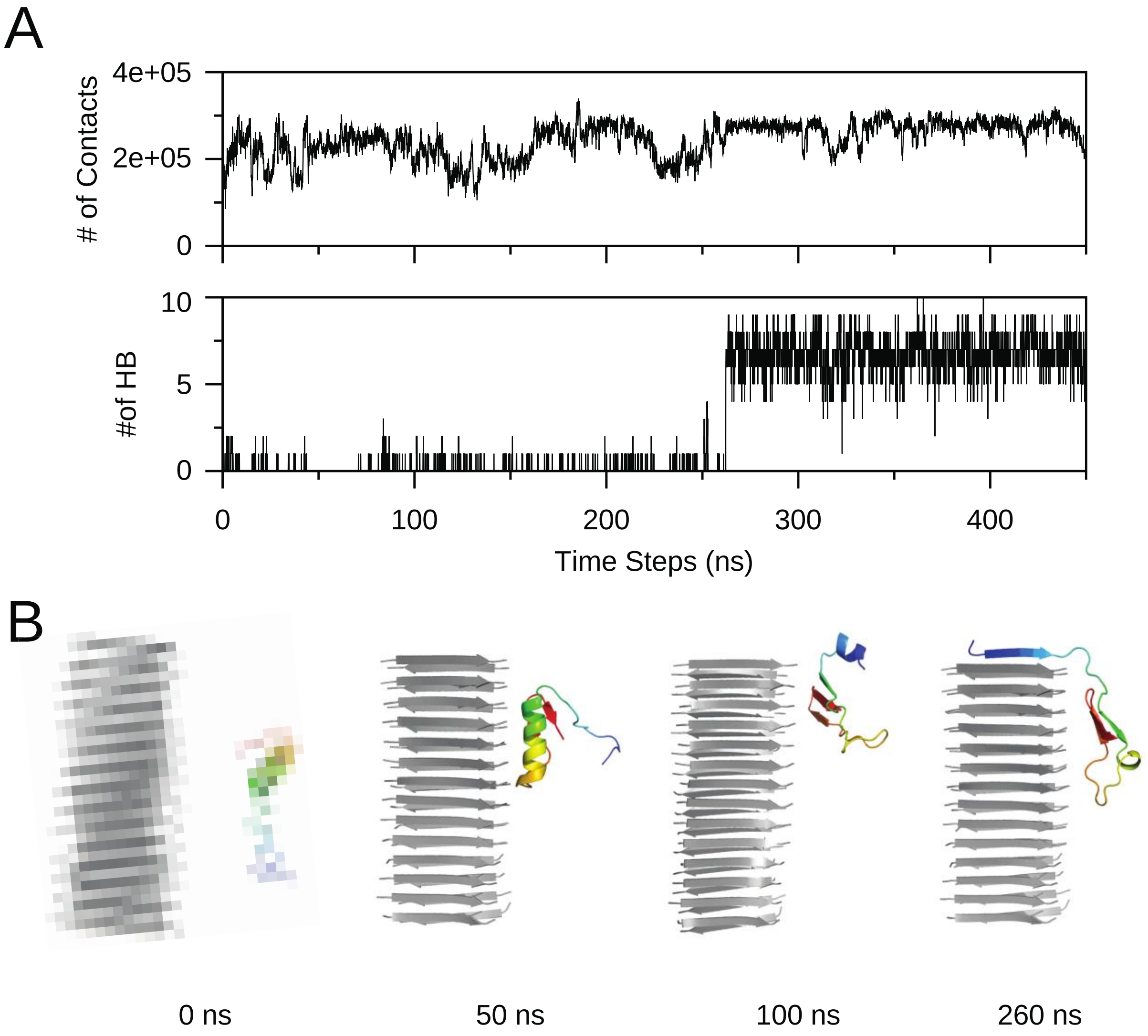

Supplementary Figure 11. (A) Time evolution of the number of atomic contacts between an Aβ monomer and a FapC nanofibril (top) and the inter-molecular H-bond backbone of an Aβ fibril (bottom) from a representative trajectory. (B) Snapshots along the simulation trajectory are shown at different times. The FapC nanofibril is shown in grey, while Aβ42 in rainbow from its N- (blue) to C-terminus (red).

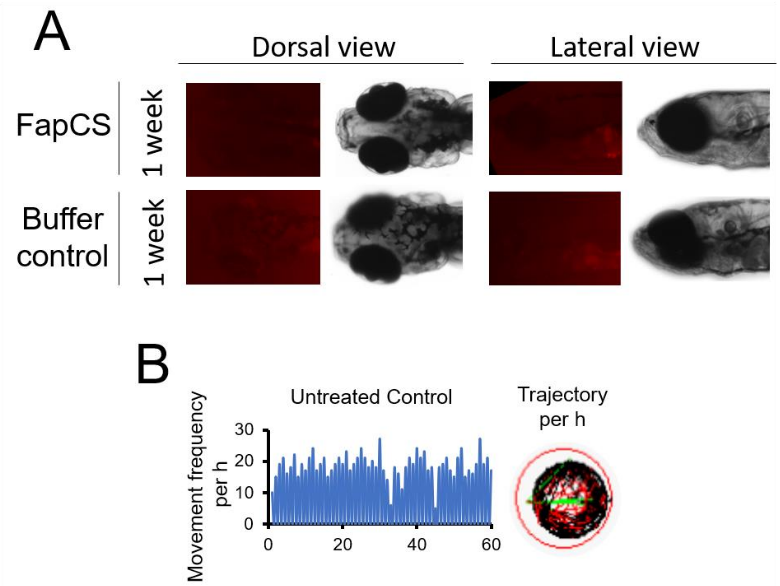

Supplementary Figure 12. (A) Whole-mount fluorescence imaging of FapCS and buffer control at the one-week time point. (B) Movement frequency and trajectory of untreated zebrafish larvae over the period of 1 h observation (n=10 per group and 3 groups per sample).

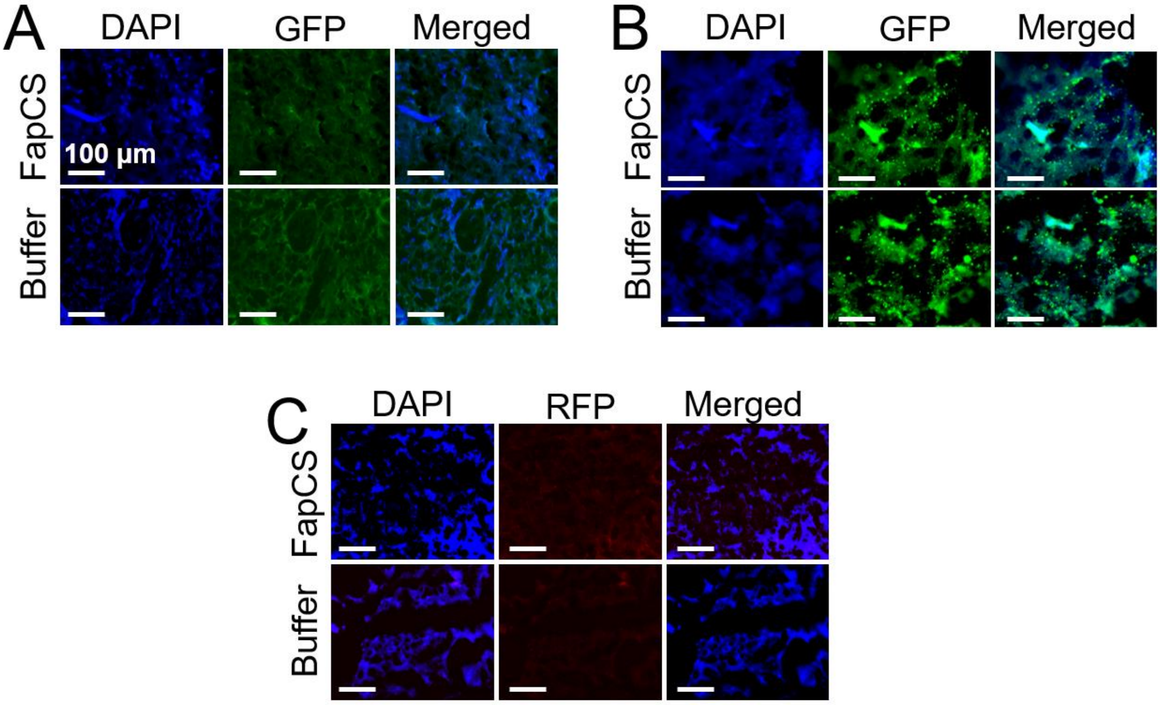

Supplementary Figure 13. Immunohistochemistry (IHC) of adult zebrafish brain slices for (A) Aβ deposition, (B) depletion of synaptophysin positive cells and (C) TUNEL assay for neuronal cell death for FapCS and buffer control.

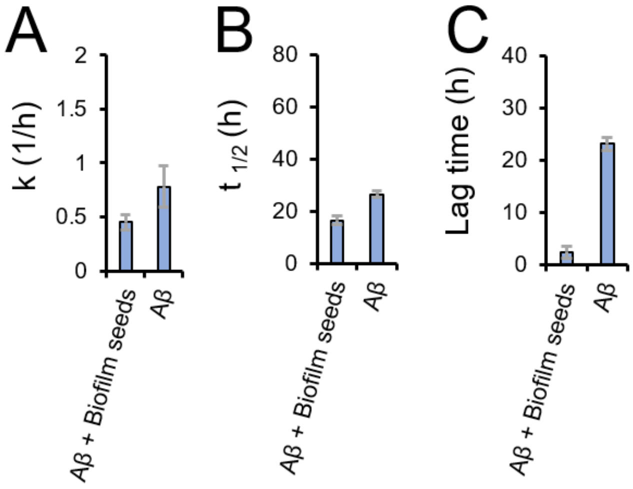

Supplementary Figure 14. ThT kinetic parameters of (A) fibrillization rate constant (k), (B) half-life $t_{1/2}$ and (C) lag time for Aβ in the presence and absence of *P. aeruginosa* biofilm fragments (n=3).

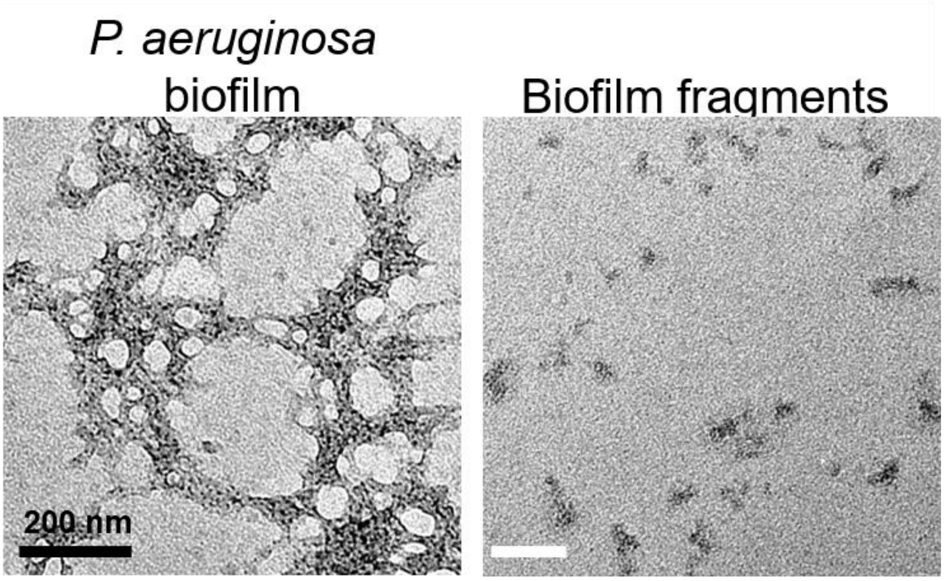

Supplementary Figure 15. TEM images for *P. aeruginosa* biofilm and sonicated biofilm fragments/seeds.
